# Order and Stochastic Dynamics in Drosophila Planar Cell Polarity

**DOI:** 10.1371/journal.pcbi.1000628

**Published:** 2009-12-24

**Authors:** Yoram Burak, Boris I. Shraiman

**Affiliations:** 1 Center for Brain Science, Harvard University, Cambridge, Massachusetts, United States of America; 2 Kavli Institute for Theoretical Physics, University of California, Santa Barbara, Santa Barbara, California, United States of America; 3 Department of Physics, University of California, Santa Barbara, Santa Barbara, California, United States of America; California Institute of Technology, United States of America

## Abstract

Cells in the wing blade of *Drosophila melanogaster* exhibit an in-plane polarization causing distal orientation of hairs. Establishment of the Planar Cell Polarity (PCP) involves intercellular interactions as well as a global orienting signal. Many of the genetic and molecular components underlying this process have been experimentally identified and a recently advanced system-level model has suggested that the observed mutant phenotypes can be understood in terms of intercellular interactions involving asymmetric localization of membrane bound proteins. Among key open questions in understanding the emergence of ordered polarization is the effect of stochasticity and the role of the global orienting signal. These issues relate closely to our understanding of ferromagnetism in physical systems. Here we pursue this analogy to understand the emergence of PCP order. To this end we develop a semi-phenomenological representation of the underlying molecular processes and define a “phase diagram” of the model which provides a global view of the dependence of the phenotype on parameters. We show that the dynamics of PCP has two regimes: rapid growth in the amplitude of local polarization followed by a slower process of alignment which progresses from small to large scales. We discuss the response of the tissue to various types of orienting signals and show that global PCP order can be achieved with a weak orienting signal provided that it acts during the early phase of the process. Finally we define and discuss some of the experimental predictions of the model.

## Author Summary

Epithelial tissues are often polarized in a preferred direction which determines, for example, the direction of hair growth on mammalian skin, the orientation of scales in fish, the alignment of ommatidia in the fly eye and of sensory hair cells in the vertebrate cochlea. This in-plane polarization, known as planar cell polarity, is one of the morphogenetic fields that play a role in tissue patterning during development. Here we focus on planar cell polarity in the fly wing, where protein localization and inter-cellular ligand-receptor interactions combine with an unknown orienting signal to establish planar cell polarity of the wing epithelium. We demonstrate an analogy between this process and models of ferromagnetism in physical systems that have been studied extensively using the tools of statistical mechanics. The analogy helps in understanding how local interactions between cells can lead to global polarization order and elucidate the role of global orienting signals and the dependence of the dynamics of the process on parameters. We demonstrate that in the absence of an external orienting signal swirling patterns should emerge due to random noise. We propose ways to test this prediction and ways to quantify the magnitude and spatial variation of the unknown external orienting signal.

## Introduction

Epithelia in diverse tissues, in addition to their apico-basal polarization, acquire a polarization within the two-dimensional layer of cells – a phenomenon called planar cell polarity (PCP) [1]–[5]. In the developing wing of *Drosophila*, PCP determines the growth direction of small hairs that extend radially from cell boundaries. In a wild-type wing, where cells are approximately hexagonal and form a regular honeycomb lattice, all of these hairs point to the distal direction.

**Figure 1:**
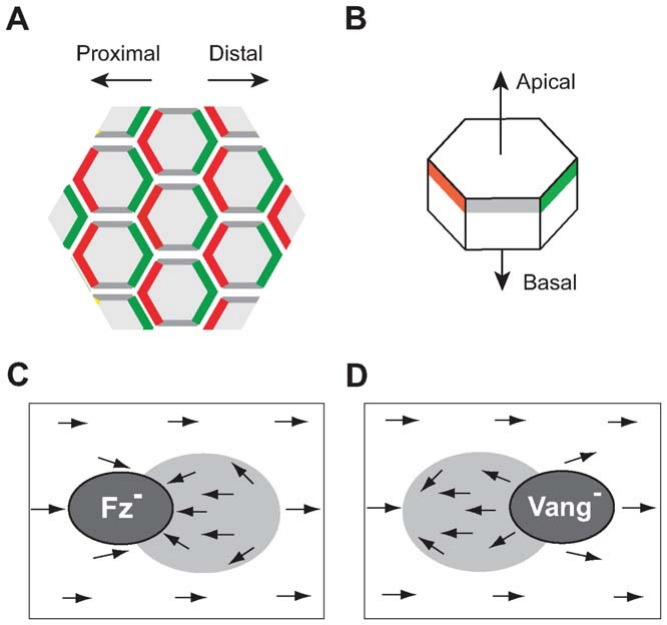
Summary of experimental observations. (A) Protein localization pattern in wild-type wing: Fz (green) localizes on the distal membrane, together with Dsh, while Vang (red) localizes on the proximal membrane, together with Pk. (B) Key PCP proteins localize apically in the adherens junction area, within a strip of about 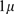 from the top [7],[11],[12],[39]. (C,D) Mutant *fz* (C) and *Vang* (D) clones influence the polarity of wild-type cells bordering the clone such that it points towards the clone (*fz*, C) or away from it (*Vang*, D). This effect is propagated to a large patch of wild-type cells that are distal to the clone (*fz*) or proximal to it (*Vang*) [40]. Over-expression of *fz* causes an effect similar to that of *Vang* mutant clones, and over-expression of *Vang* causes an effect similar to *fz* mutants.

A series of recent experiments show that several key proteins [6], including the transmembrane proteins Frizzled (Fz) and Van-Gogh (Vang) and the cytosolic proteins Dishevelled (Dsh) and Prickled (Pk), localize asymmetrically on cell boundaries [7]–[12] - defining a direction in the plane within each cell and forming a characteristic zig-zag pattern of protein localization on the lattice (Fig. 1A).

Other experiments show that local PCP orientation depends on inter-cellular signaling. First, mutant clones in which *fz* or *Vang* activity is suppressed or amplified, cause characteristic and reproducible inversion of polarity in large patches of cells that are proximal or distal to the clone [13]. These observations are summarized in Figs. 1 C,D. Second, in *fat* mutant clones [14],[15] hairs do not all point correctly in the distal direction, yet, their orientation is strongly correlated between nearby cells and varies gradually across the tissue creating a characteristic swirling pattern.

Thus the experimental evidence suggests that an interaction between neighboring cells tends to locally align their polarity [1],[3],[14]. This local polarity need not point distally unless, in addition, there is a global orienting signal that picks out the distal direction throughout the wing (most likely originating with the Dpp morphogen gradient which defines the Anterior-Posterior axis of the wing in the larval stage of development [16]). Yet, aside from a clear involvement of protocadherin *fat* [17],[18] the molecular details of this pathway remains for now unknown. The swirling patterns in *fat* mutants [14] and recent evidence [15],[19], suggest that the orienting field is related to the presence of a “gradient” in the *fat*, *four-jointed*, and *dachs* pathway.

These observations evoke an analogy between PCP and the behavior of ferromagnets, extensively studied in physics and well understood in terms of statistical mechanics of relatively simple models [20]. In these models each atomic site is assigned a magnetic dipole – spin – which can assume a different orientation (analogous to the direction of polarization in an epithelial cell). The salient properties of ferromagnets arise from the opposing influence of an interaction between neighboring spins, which tends to co-align their orientation, and the influence of thermal fluctuations, which tend to randomize the spin direction. Ferromagnets typically exhibit two phases of behavior: a high temperature phase, where spins are disordered and a low temperature ferromagnetic phase, where the interactions dominate over thermal fluctuations – leading to a spontaneous polarization in an arbitrary direction. In this state even a small external magnetic field has a big effect on magnetic polarization as the spontaneous polarization aligns itself with the external field, yet the dynamics leading to global alignment can be quite slow.

An essential lesson from statistical mechanics is that the ordered and disordered states exist in a broad class of models and can be discussed in a general context, focusing on a classification of the different regimes as a function of a few parameters. We follow this lesson by focusing the study on the competition between the intercellular interaction and the disordering influence of the fluctuations introduced by the noisy molecular interactions. As in statistical mechanics we define a phase diagram which identifies different regimes of behavior in the space of the most relevant parameters. We then address the role of the global directional signal in the dynamics of global alignment.

A molecular model for PCP formation was recently proposed in Ref. [21], and was shown to reproduce a number of experimental findings. This model involves 38 parameters that were adjusted to successfully reproduce a set of wild-type and mutant phenotypes. Here we pursue an alternative approach and instead of moving on to more and more complex models develop a model with a smaller number of degrees of freedom and a smaller number of parameters. Instead of fixing a particular set of parameters by fitting the data we explore the generic behavior of the model as a function of parameters defining quantitative features characteristic of the different phases. In formulating the model we identify several essential ingredients, required to obtain the characteristic zig-zag pattern and the non-autonomy of *fz* and *Vang* mutant clones. We expect our simplified model to capture important properties of PCP, although it does not incorporate all the molecular details.

After discussing the essential ingredients of the model, we obtain a phase diagram describing its steady state properties. We then consider the dynamics of local polarization strength and orientation in the absence and in the presence of a global orienting signal. We show that global alignment can be achieved with a weak global orienting signal provided it is present throughout the tissue at the earliest stage of PCP dynamics. Finally we discuss the experimental predictions coming out of the model and the tools required to test these predictions.

## Results

### Model ingredients

Three essential ingredients are included in the model, to account for the characteristic zig-zag patterns of protein localization and for the non-autonomy of *fz* and *Vang* mutant clones.

#### Two membrane proteins form complexes across the inter-cellular interface

As in Ref. [21] we assume that two membrane-bound proteins, 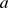 and 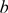 – standing for Fz and Vang - form complexes across inter-cellular interfaces. This is the source of intercellular interaction in the model.

Complex formation across cell interfaces accounts in a simple way for the non-autonomous effect of clones in which either 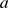 or 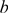 are mutated. However to account for the observed localization of Fz and Vang proteins on the *opposite* sides of the cell interface there must be a mechanism which prevents 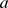, 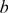 (or Fz and Vang) from mingling with each other on the same side of the interface. Thus the next two assumptions introduce molecular interactions acting inside each cell, leading to spontaneous segregation of the complexes and driving the protein distribution towards a non-uniform state.

#### Complex formation on a single inter-cellular interface is bistable

**Figure 2:**
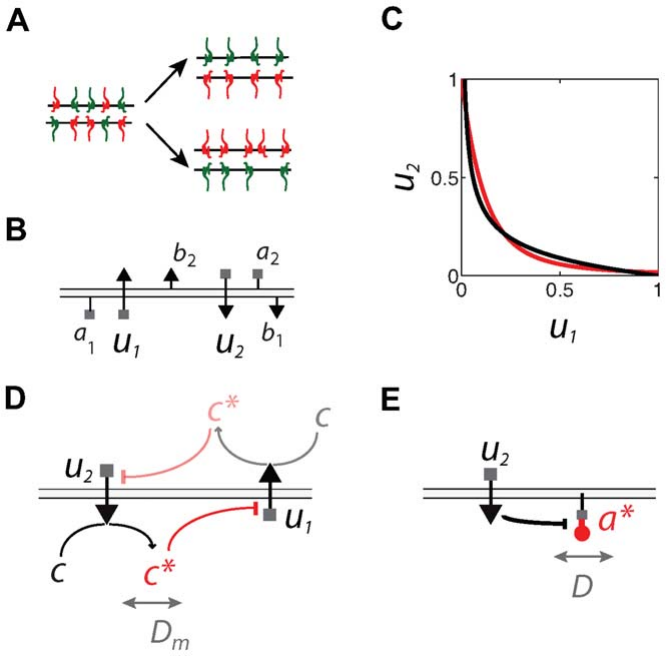
Key model ingredients. (A) Bistability on an interface. (B) Notation used for 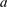
−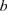 complex binding and unbinding, Eqs. (1)–(2). (C) Nullclines for 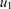 and 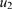 (red and black lines), exhibiting an unstable fixed point with 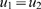 and two symmetry-breaking stable fixed points. (D)–(E) Possible mechanisms for the generation of a non-local field, based on the modification of a diffusible protein: (D) A cytoplasmic messenger protein is modified when it meets the 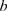 side of an 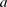
−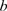 complex. It then continues to diffuse and, when it meets the 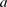 side of a complex it promotes its unbinding. (E) Instead of modifying a separate messenger protein, the 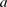 protein is directly modified by binding a cytoplasmic protein; 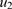 complexes locally affect the fraction of modified 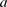 proteins and this, in turn, affects their affinity for forming complexes with 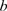-s on the opposite side of the interface.

We assume that complexes of one polarization (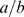) inhibit formation of complexes of the opposite polarization (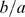), and that this inhibition leads to bistability at the level of a single interface between two cells (Fig. 2A). As a simple example, consider the following dynamics of complex binding and unbinding on a single, planar interface (Fig. 2B),
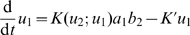
(1)

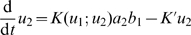
(2)where 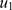 and 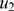 represent concentrations of interfacial complexes with two possible polarizations (respectively 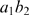 and 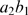) and 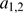, 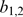 are concentrations of free (unbound) proteins on the two sides of the interface.

The positive and negative feedback on complex formation is represented through the dependence of the rate coefficients 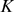 on 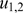 (see Methods). E.g., enhancement of 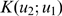 in Eq. (1) with increasing 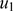 or suppression with increasing 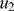. If this dependence is sufficiently non-linear, the dynamics lead to two stable steady states: one with 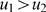, the other with 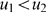, as illustrated in Fig. 2C. Feedback effects could be equally well modeled by an opposite modulation of 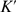 and in reality quite likely involve modulation of both 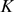 and 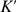. As an example, consider the case where there is only negative feedback through the dependence of 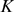 or 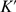 on 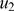. If the free 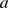 and 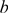 diffuse sufficiently rapidly, 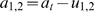 and 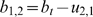, where 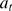 and 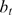 are the total available concentrations of 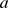 and 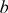 proteins. It is then easy to see that for bistability 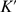 or 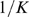 must be a convex increasing function of 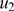.

#### Inhibition acts non-locally within each cell

While bistability of the complex formation would suffice to explain localization of Fz and Vang on the opposite sides of each interface between cells, in order to explain segregation of Fz and Vang to the opposite sides of each cell we assume that the mutual inhibition of 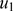 and 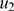 complexes acts non-locally within a cell. Hence instead of making 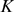 in Eqs. (1–2) be a local function of 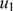 and 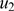 we assume that 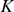 is a function of 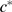, the concentration of a messenger molecule which is itself a non-local function of the 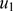 and 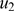 distribution over the surface of a given cell. The messenger molecule thus mediates an interaction between 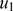 and 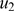 complexes, *i.e.*, 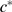 diffuses *within* each cell creating an effective repulsion between 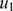 and 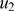 complexes on adjacent interfaces. The non-local repulsion will for a broad range of parameters result in a dipole-like distribution of 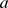 and 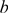 (and hence of the 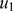 and 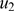 complexes) over the surface of each cell.

A plausible and quite general mechanism for generating such a non-local inhibitory signal involves the modification of a diffusible protein, as illustrated in Figs. 2 D,E where we denote the unmodified and modified protein by 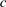 and 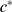, respectively. The rate of modification 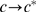 at a given point on the membrane depends on the local density of 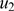 complexes and information is transmitted within the cell by diffusion of the modified protein. Many variations on this general theme are possible and are discussed in detail in the supporting analysis (Text S1, Part I). Below we follow the scheme shown in Fig. 2E, where the membrane-bound protein 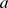 serves the role of the messenger protein 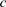. Modification of 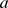 corresponds to the binding of a cytoplasmic protein, and this process is inhibited by 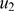 complexes. The fraction 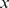 of unmodified proteins then obeys the equation
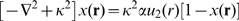
(3)where 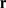 is the location on the membrane. The parameters 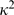 and 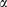, related to the rate constants for modification of 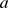 are discussed in the supporting analysis (Text S1, part I). Note that increase of 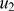 increases 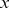 and that the influence of 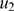 is non-local, with a characteristic range set by 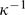. Finally, we assume that only modified 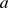 proteins can form complexes, hence the rate coefficient 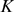 is proportional to 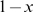 (Eq. 7). Other details of the non-local inhibition mechanism are described in *Methods*. Interestingly, we find that to maintain a non-local signal in a steady state an energy flux is necessary (Text S1, part I).

### Stochastic dynamics

There are several reasons why the dynamic equations are not deterministic. Even in the steady state, interfacial complexes not only bind and unbind due to thermal fluctuations, but like nearly everything else inside the cell are being constantly recycled and reassembled. Stochastic fluctuations arise from the molecular noise of reactions and the variability in the state of the cell defining the “intrinsic” and “extrinsic” noise [22]. It will suffice however to describe stochasticity of complex binding and unbinding as if it were a Poisson process. Equation (1) is thus replaced by a stochastic equation,
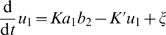
(4)[and a similar modification applies to Eq. (2)] where the noise 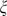 can be approximated as white Gaussian noise if the number of molecules per cell is not too small. Assuming that the dominant contribution comes from the finite number of molecules participating in the binding/unbinding dynamics, the variance of 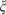 is inversely proportional to 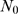 (see *Methods*), where 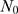 is defined as the number of 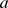 molecules per interface: 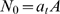 where 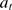 is the total concentration of 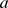 molecules (bound and unbound) and 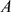 is the area of an interface (about 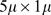 – see Fig. 1B). Since the variance of 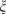 decreases with increase of 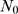, 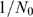 plays a role similar to temperature in a ferromagnet. If there are 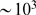 Fz molecules per cell [23], 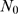 is of order 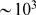 resulting in the root-mean-square fluctuations of the order of 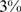 (i.e. 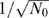) of the mean.

Other sources of intrinsic noise, in addition to the stochasticity of binding and unbinding events, may increase the noise variance beyond the above estimate. These additional noise sources include, for example, stochasticity in the signaling pathway that generates the non-local inhibition within each cell, or fluctuations in 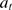 and 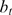. Such sources of intrinsic noise, acting upstream of 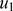 and 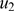, are propagated to the PCP signaling dynamics through the dynamics of complex formation, and can thus be described qualitatively by the noise term in Eq. (4), with an effective value of 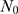 that is possibly smaller than predicted from the number of 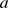 and 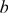 molecules alone.

### Phase diagram

**Figure 3:**
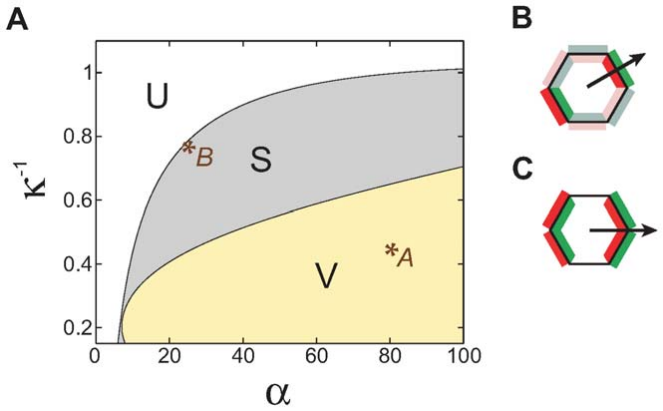
Phase diagram in the deterministic limit. (A) Phase diagram in the deterministic limit, dissected in the 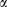-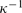 plane. The other parameters are 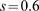, 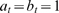, and 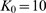. Crosses designate the two loci, A and B, used in the numerical simulations shown in Figs. 4,5, and 6. (B) Steady state in region S: polarity points towards a side. (C) Steady state in region V: polarity points towards a vertex. In region U protein distribution is unpolarized.

What are the consequences of the model defined above when cells are arranged on a hexagonal lattice? Let us first consider the steady state in the deterministic limit. Fig. 3A shows a typical phase diagram on a two-dimensional plane dissecting our five dimensional parameter space (see *Methods*): the 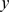 axis is the range of the non-local interaction in units of the cell lattice spacing, and the 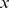 axis the coefficient 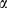 which controls inhibition (see *Methods*).

In the region labeled 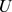 there is a unique steady state in which there is no polarization of the protein distribution. In contrast, in region 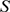 the stable steady state has the symmetry shown in Fig. 3B: Both 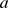 and 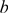 distributions carry a vector dipole moment that points towards the center of a side, and due to the lattice symmetry there are six equivalent states of this type. A uniform steady state exists as well, but it is unstable. Region 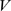 differs from 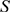 in the direction of the dipole, which points towards a vertex instead of pointing towards and edge (Fig. 3C).

The transition from the uniform state, 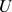, to the edge state, 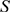 in the phase diagram is continuous: the dipole moment tends to zero when approaching the phase boundary from the 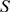 side. A similar transition from a 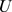 state to a 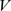 state can exist as well, and is present on another two dimensional “slice” through the parameter space of our model. This transition is also continuous.

**Figure 4:**
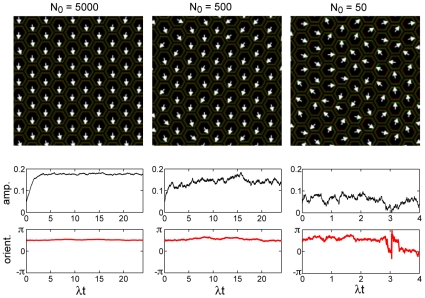
Stochasticity: ordered and disordered states. Ordered and disordered states under stochastic dynamics. A lattice containing 1840 cells (as in Fig. 5) is initiated with all dipoles pointing in the downwards direction. Stochastic dynamics are then followed to assess whether long range order in the cell array is maintained, and this is done for several different values of 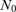, the average number of molecules per interface. While long range order is maintained for 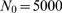 and 500 (left and center panels), long range order is destroyed by the stochastic fluctuations for 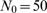 (right panels), as quantified by the center panels which track the dynamics of the polarization averaged over all cells (Center panels, amplitude: 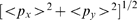; Bottom panels, orientation). The top panels show a snapshot of a subset of cells at the end of the simulation.

We next consider the effects of stochasticity, which were ignored in the discussion above by setting 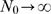. When 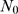 is finite (similar to a non-vanishing temperature in a spin model), we ask whether the steady state maintains long-range order: i.e. whether a particular orientation is singled out throughout the lattice and the dipole moment has a non-zero average. In the language of the analogy with magnetic systems this would be a ferromagnetic state. The latter disappears as the temperature increases above a certain critical value, giving way to a paramagnetic state where dipole moments point in random directions and the average polarization vanishes (an intermediate state with quasi-long range order may exist as well, in similarity to 2-dimensional clock models [24]–[27]). Hence, we expect an ordered state to be stable only when 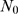 is sufficiently large, and this is indeed observed in our simulations (Fig. 4). Yet with a realistic number of molecules per cell, in the order of several thousands, the vertex and side states in our model are typically ferromagnetic.

It may thus appear that when 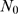 takes realistic values the system is in an ordered state and stochasticity is altogether unimportant. However, as we discuss next, the steady state is not necessarily reached within the time scales of wing development, and stochasticity plays an important role in the dynamics of ordering.

### Dynamics of ordering in the absence of a global orienting signal

**Figure 5:**
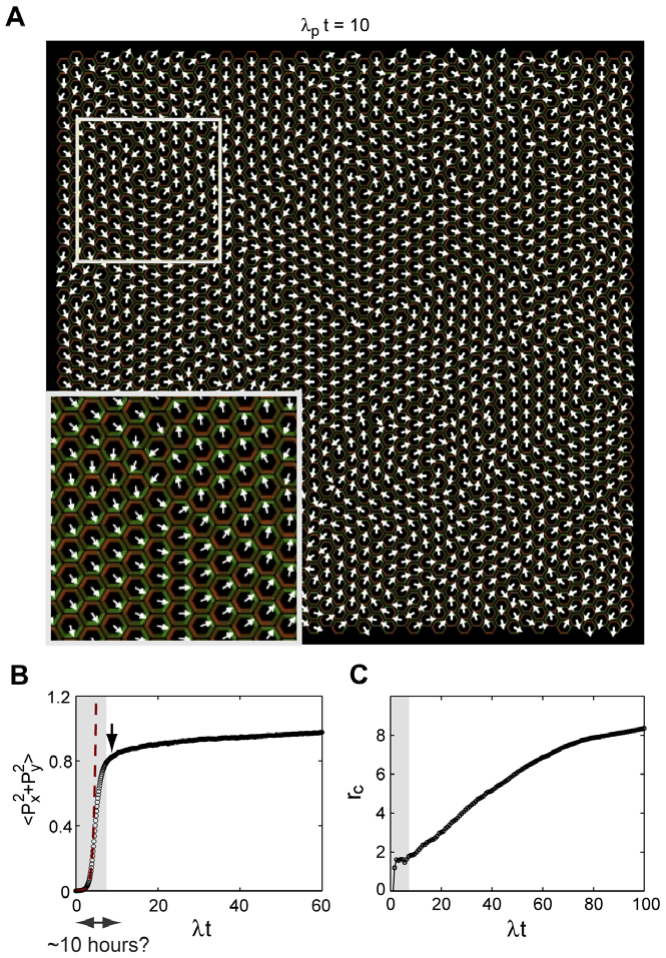
Stochastic dynamics in the absence of an orienting signal. Results from a stochastic simulation in locus A of Fig. 3A with 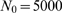 and with no orienting signal, starting from the uniform steady state. The lattice contains 1840 hexagonal cells tiling a square region with periodic boundary conditions. (A) Pattern of polarity orientation shortly after amplitude saturation, at 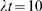. Arrows point in the direction of the dipole moment. Inset: Close-up. Green and red represent 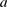 and 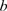 concentration, respectively. (B) Average square amplitude of polarity as a function of time. Arrow marks the time shown in panel A. Dashed line: Eq. (5). (C) Measure of the correlation length as a function of time, 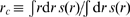 where 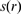 is the radial correlation function 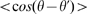.

Let us consider the dynamics of PCP formation, first in the absence of a global orienting signal. Fig. 5 shows results from a stochastic simulation, starting from a state where 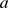 and 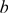 are uniformly distributed in all cells.

We can identify two stages of the process. The first stage corresponds to a gradual build up of a dipolar polarization on the cellular level. The dipole initially points in a random direction, but as its amplitude increases with time (Fig. 5B) local polarization begins to re-orient. At the end of this stage, when amplitude saturates, there is no global choice of PCP direction, but the orientation of nearby cells is strongly correlated: as an example, Fig. 5A shows the configuration of dipoles shortly after saturation. The second stage, which follows amplitude saturation, exhibits slow coarsening dynamics [27]: polarity direction is approximately aligned within discrete domains, the size of which gradually expands by movement of their boundaries. Note also the existence of vortex-like defects [28] (Fig. 5A and Fig. S1). Coarsening ultimately leads to a spatially uniform steady state, but this process occurs over a long time scale compared to that of amplitude growth.

A quantitative theory of the early dynamics is obtained from the linear instability of the uniform steady state (described in detail in Text S1, part II). The variance of the local dipole amplitude increases exponentially in time with a characteristic time scale 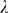,
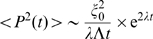
(5)where for simplicity numeric prefactors of order unity are omitted (see Text S1, part II). In this equation 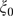 is the amplitude of noise in the unstable uniform steady state, and both 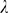 and 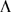 are found from the instability analysis (Text S1, part II). This prediction is shown in Fig. 5B (dashed line) for comparison with the simulation.

Two additional insights come from the analysis of early dynamics (Text S1, part II). First, PCP is initially isotropic, despite the discrete 6-fold symmetry of the hexagonal cell lattice. Consequently, the dipole moment initially has equal probability to point in any direction in the interval 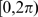. Second, the spatial correlation established during the early dynamics typically has a longer range in the direction parallel to the dipole, compared to the perpendicular direction. These two properties of the dynamics lead to a characteristic swirling pattern before non-linearities set in. The range of correlation at this stage depends on the location in the phase diagram and increases logarithmically as a function of 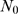.

### Effect of global orienting signals

We next consider how various types of symmetry-breaking orienting signals influence PCP dynamics.

#### Boundary orienting signal

For example, a row of cells that do not express 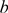 (or, alternatively, 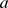) can serve as a boundary orienting signal. Can such a signal orient a tissue as large as the wing? In a deterministic model without any stochasticity, the boundary is the only cause for symmetry breaking, and will necessarily set polarity orientation throughout the tissue. In the presence of noise a local choice of polarity is established in the bulk of the wing, and competes with the boundary signal.

**Figure 6:**
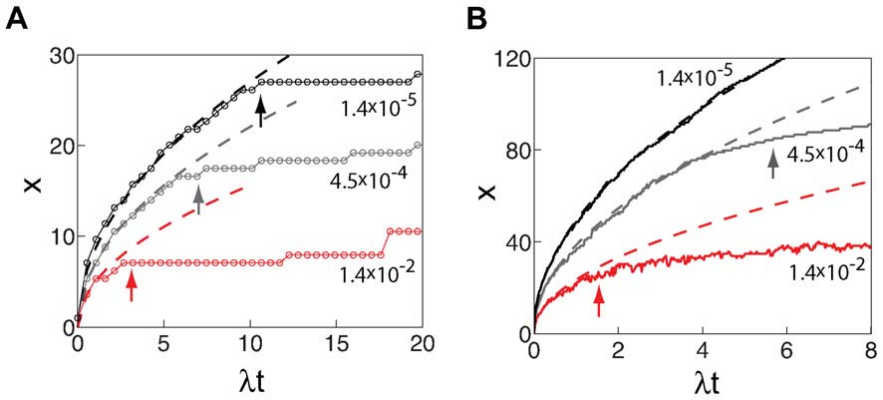
Response to an orienting signal at a boundary. Position of the front 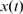 during a stochastic simulation. The front location is defined as the most distal position such that all cells proximal to this position have their PCP dipole pointing distally (*i.e.*, the dipole has a positive projection in the proximal-distal direction) and is shown in units of 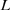, the distance between neighboring cells. Simulations were run on a honeycomb lattice with rectangular boundaries, extending 200 cells sizes (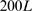) in the direction of front propagation (the proximal-distal axis), and 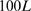 in the perpendicular direction. (A) Locus A in the phase diagram of Fig. 3A, far from the phase transition. The red trace corresponds to a realistic number of molecules per cell, 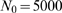, and to stochastic noise 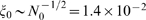. The other two traces correspond to higher, non-realistic values of 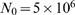 (gray) and 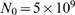 (black). Dashed lines show the prediction of Eqs. (S49)–(S50), where 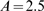 in all traces was estimated from the polarity amplitude near the boundary in the beginning of the simulation, and the numbers next to each trace represent 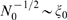. The arrows designate the time of amplitude saturation in the bulk, estimated from Eq. (S44) of the supporting analysis (Text S1). Note that after saturation front propagation is slowed down considerably. (B) Similar plots obtained from locus B in the phase diagram. Proximity to the phase transition increases the range of front propagation [as seen from comparison with (A)], but even here an unrealistically small amount of noise is required to reach a propagation range comparable to the wing size. This is due to the weak, logarithmic dependence on 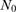 (through the value of 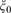) in Eq. (S50).

During amplitude growth a moving front separates two regions of the tissue: between the boundary and the front all cells point in the orientation set by the boundary, whereas beyond the front cells point in all possible directions. The position 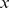 of the front increases sub-linearly with time, 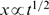, with a prefactor that depends on the position in the phase diagram and increases logarithmically with increase of 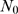 (Fig. 6 and Text S1, part II). After amplitude saturation, when an independent choice of polarity is established in the bulk, front propagation is arrested (more precisely the front continues to diffuse, but this occurs over a much longer time scale than the initial propagation). Our simulations of this process for different parameters (see Fig. 6) suggest that a boundary induced polarization would not reliably spread across hundreds of cells on a plausible time scale.

#### Bulk orienting signal

Any perturbation that breaks the symmetry of forming 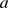
−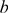 versus 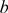
−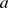 pairs can potentially act as a bulk signal. Symmetry breaking can occur, for example, through a graded expression of 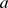 or 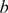 proteins in the tissue, or alternatively, another protein with a graded distribution might sequester or hyper-activate either 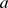 or 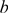. Such graded distributions may be expected to arise from morphogen gradients. Yet, the asymmetry on the level of a single cell, due to such an effect is expected to be weak because the concentration gradient of the protein is small on the scale of a single cell. On the other hand, a bulk magnetic field of any magnitude will eventually orient an ordered ferromagnet. In the PCP context, with the developmental time scale of 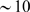 hours corresponding to the PCP amplitude growth stage, an important question to ask is whether a weak bulk field can orient the whole wing within this limited time frame.

To address this question we focus on a particular type of a bulk orienting signal that can be easily quantified. A graded expression of 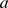 (or 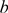) within the wing acts as a signal that orients polarity in parallel to the gradient direction. The effect of such a field can be analyzed analytically and is described in the supporting analysis (Text S1, part II).

In our model, a gradient in 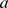 expression corresponding to a 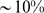 change across the wing (assuming that the wing is 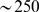 cells across and a 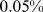 change between adjacent cells) yields full orientation in the distal direction before amplitude saturates in state 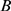 of the phase diagram (Fig. 3A) with 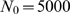. A signal ten times larger, which corresponds to a two-fold change in concentration across the wing, is sufficient to achieve full orientation in state A, which is far from the phase transition. These results suggest that a weak orienting signal (e.g. a 0.1% per cell variation in protein level) can effectively orient the wing within the time scale of about 10 hours.

**Figure 7:**
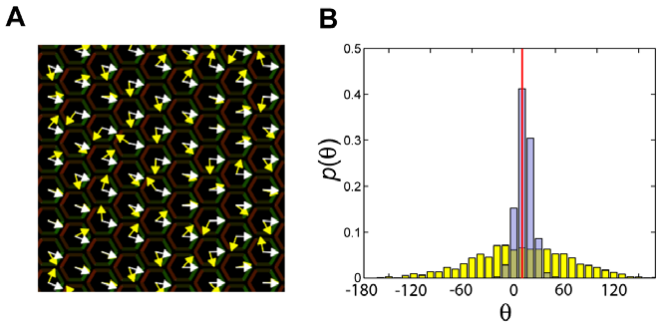
Smoothing of a noisy orienting signal. Response of PCP orientation to a noisy orienting signal present in each cell. The direction of the signal (yellow arrows in panel A) is uncorrelated in different cells and is biased towards the direction designated by the red vertical line in panel (B) (roughly the distal direction), but is widely distributed in the range 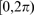 (A) The PCP response (white arrows) is shown from a stochastic simulation in locus A of the phase diagram (Fig. 3A) with 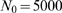, at 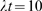. (B) Distribution of PCP orientation at 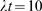 (gray bars) compared to the distribution of orientation of the orienting signal (yellow bars).

It is possible that the orienting signal is not derived directly from a protein gradient: An early polarization may exist in each cell before the asymmetric localization of the key PCP proteins develops. For example, an early polarization exists in the distribution of Widerborst [29],[30]. In addition, recent evidence [19],[31] suggests that proteins in the *dachs*-*fat*-*daschous* pathway are asymmetrically distributed as well. Such an early polarization, of a protein other than Fz and Vang, may be a rough readout of a morphogen gradient, and may couple to the dynamics of PCP proteins to establish an orienting signal. In this case the cell-cell interaction in PCP may serve to smooth such a signal, creating a readout that is more spatially-uniform and accurate than the input present in each cell alone. This is demonstrated in Fig. 7, where a noisy orienting signal (yellow arrows) is compared to polarity response (white arrows).

## Discussion

### 

#### Under-expression of *fz* and *Vang*

The effect of changing particular parameters of the model may depend on the position within the phase diagram. An example can be seen in the lower-left part of the diagram in Fig. 3, where increasing the parameter 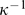 could either switch from a vertex state to a side state, or vice versa. However, we find quite generally that decreasing 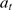 or 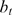 causes a transition to the uniform state. Reducing protein numbers in the cell corresponds in our model to a simultaneous decrease in 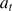 or 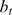, and in 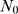 which tends to destabilize the ordered state. Hence within our model a decrease in Fz and Vang concentrations increases deviations of hair polarity from the correct distal direction and eventually destroys the ordered state altogether. While our model is in agreement with the broad effect of *fz* mutation or under-expression, it also generates new and quantitative predictions, as discussed below.

#### PCP dynamics

The observed asymmetry in distribution of Fz and Vang builds up gradually over a time scale of about ten hours, between 18 and 32 hours after puparium formation [7]–[12]. The simplest interpretation of this observation is that PCP formation takes place during the first stage of the dynamics, before amplitude saturation. The characteristic time scale 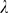 should then be of order 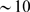 hours. An alternative scenario is that a local dipole moment builds up in each cell on a much shorter time scale, and that PCP dynamics occurs mostly within the second stage of domain growth. In this latter scenario we expect to observe domains in which polarity points to directions other than the distal one. Since existence of such domains in not reported experimentally, the evidence appears to support the first scenario. Experimental observations were made mostly from static images in which proteins on the two sides of each interface could not be resolved. It will thus be extremely useful to quantify the dynamics of PCP amplitude and orientation, in order to distinguish unambiguously between the two scenarios. Such quantification would make it possible to test the detailed predictions on dynamics.

#### Swirling patterns in the absence of an orienting signal

The model predicts that swirling patterns should emerge in the absence of an orienting signal. These patterns are consistent with those observed in large *fat* mutant clones, at least qualitatively. The prediction thus supports the hypotheses that *fat* mutants lack a coupling with the orienting signal [14]. However, *fat* mutants differ from wild type tissues in an important way, namely, that their cell arrangement is less ordered than in wild type tissues [15]. There are thus two possible mechanisms leading to disorder in *fat* mutants: one arising from the role of stochasticity in the absence of an orienting signal, and the other arising from lattice disorder. These two mechanisms are not necessarily mutually exclusive.

Regardless of the mechanism at work in *fat* mutants, our model predicts that even in an ordered lattice without excess defects, swirling patterns will appear in the absence of an orienting signal, followed by slow coarsening dynamics. We envision three potential ways to test this hypotheses. First, if *fat* is necessary for the coupling with an orienting signal, but also plays a separate role in lattice repacking, disabling *fat* activity at a sufficiently late stage of the dynamics, after lattice repacking [32], may inhibit coupling with the orienting signal without influencing lattice order. Second, it may be possible to find other mutations in which lattice order is not disrupted, but the coupling with the orienting signal is absent. Third, it may be possible to negate the effect of the endogenous distally orienting signal by inducing an orienting signal in the proximal direction.

#### An artificial bulk orienting signal

We predict that graded expression of 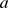 or 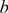 will act as an orienting signal. This prediction is consistent with experiments in which a gradient in expression of *fz* was induced using heat-shock promoters, causing inversion of hair-growth direction [33]. A similar effect is expected with a graded expression of *Vang*.

Inducing a gradient of Fz or Vang protein concentration may provide a way to cancel the endogenous signal in order to test the predictions discussed above. Further, inducing such a gradient in a tunable, quantifiable manner could be a realistic experimental objective, *e.g.*, using light-switchable promoter systems that allow precise spatio-temporal control of gene expression [34]. In addition to testing the prediction that 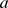 and 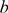 gradients can act as an orienting signal, measuring the magnitude of gradients that induce a significant perturbation in the PCP pattern can provide a way to quantify the magnitude of the endogenous orienting signal and its spatial variation within the wing. Another prediction that could potentially be tested along these lines is that applying an orienting signal only at a late stage of the ordering dynamics will have only weak influence on polarity (see Fig. S2). Finally, inducing an orienting signal in *fat* mutants could help distinguish between the role of lattice disorder and the role of uncoupling from the orienting signal.

While gradients in *fz* or *Vang* expression could be used as a tool to perturb PCP in a controlled manner, experimental evidence suggests that the endogenous orienting signal is not due to a gradient in *fz* expression [23],[33],[35],[36]: First, a graded expression has not been observed experimentally (although our model suggests that very weak gradients may be sufficient to select the distal orientation). Second, uniform expression of a *fz* transgene with a heat-shock promoter is sufficient to rescue a null *fz* genotype.

#### Bulk vs. boundary signals

Our results demonstrate that a weak concentration gradient within the tissue can produce a reliable response, although the concentration change on the scale of individual cells is very small. The reliable response is achieved by the collective dynamics of the network, which effectively integrates the orienting signal over a region of the tissue larger than the size of an individual cell. Hence our results suggest that in the PCP pathway inter-cellular interactions within the network of cells serve to increase the fidelity of response to a morphogenetic field.

In contrast to the precise readout of a weak bulk signal, a boundary signal cannot effectively propagate in our model over a large number of cells. This result is expected to hold in any model that shares a fundamental aspect with our model, namely, that the uniform state is unstable and gives rise spontaneously to a patterned state driven by noise even in the absence of a global ordering field. In contrast, in an excitable system where the uniform state is stable, it may be possible to achieve patterning by a propagating front – as observed, for example, in the morphogenetic furrow during drosophila eye development [37].

#### Phenomenological models in Biology

Modeling in Biology tends to emphasize molecular detail. Yet in biological networks that involve more than a few components the typical situation is that many details are unknown, and it is imperative to devise an approach that can be insightful and predictive even in the absence of complete knowledge. Our strategy was based on building a semi-phenomenological model which attempts to identify the key microscopic aspects (e.g. formation of trans-cellular heterodimer complexes), build a simple model which parameterizes the many unknowns and systematically identify different regimes of behavior as a function of parameters (e.g. via a phase diagram). We then focus on identifying the observable effects that can help to discriminate between different regimes of the model. For example, the dynamics of intracellular polarization and the “coarsening” dynamics that extends local correlations into a global order, are identified as informative quantitative phenotypes deserving careful experimental study. The study is obviously incomplete, as it does not explicitly identify all relevant genes and molecules, but it provides a useful framework allowing to classify phenotypes and accordingly group observed genetic perturbations, and eventually refine the model at an increased level of molecular precision.

## Methods

### 

#### Model

We considered two mechanisms for establishing a non-local inhibitor field in each cell, and results were similar in the two. The examples used in the manuscript use one realization of the model which is summarized below, while a full description of both mechanisms is provided in the supporting analysis (Text S1, part I).

#### Fast diffusion

We assume that protein diffusion is fast. The relevant time-scale in this context is the typical time for diffusion of a membrane protein from one side of a cell to the opposite side, which (assuming a diffusion coefficient 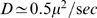) is of the order of 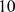 minutes. In comparison, the asymmetric pattern of protein localization arises on a time scale of several hours, so that separation of time scales appears to be a reasonable approximation. Assuming fast diffusion, free 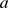 and 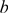 concentrations are uniform in each cell. Similarly, diffusion of bound 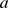/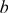 complexes equilibrates their concentration on any given interface. Complexes, however, cannot diffuse from one interface to another without unbinding first, and reforming with new constituents – processes that we assume are slow. Hence complex concentrations can vary between interfaces belonging to the same cell and the dynamics do not necessarily lead to a uniform steady state. For a regular hexagonal array of cells one then needs to keep track of six variables per cell representing the total numbers of interfacial complexes 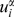 (with 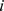 labeling the sides of cell 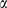).

#### Dynamic equations

The dynamics of complex concentration 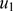 at the interface between two cells (Fig. 2B) are described by Eq. (4). The concentrations of unbound 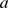 and 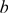 are
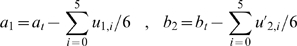
(6)where 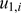 are concentrations of complexes on the six sides of cell 1, having an 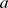 within that cell and 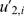 are concentration of complexes in cell 2 with a 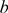 in that cell, and where 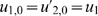 (Fig. S3). The rate K′ is a constant that we set to unity by rescaling time, and 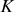 is given by
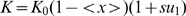
(7)where the term 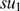 describes self-excitation, and 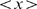 is the average on the interface of the non-local field 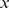, which represents the fraction of unmodified 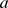 proteins. This field obeys Eq. (3), in which 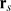 is a one-dimensional coordinate ranging from 0 to 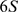, 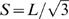 is the length of a cell side, and 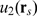 is a step-wise uniform function equal to 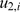 on each of the sides of cell 1.

All the parameters in this equation are dimensionless: we re-scale all concentrations by the total concentration of 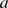 proteins, so that 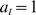. Lengths are rescaled by setting 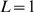. The independent parameters in the model are thus 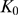, 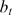, 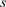, 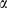, 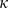, and 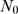. The parameters of states A and B, as well as the value of 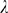 in these states are summarized in Table 1.

**Table 1:** Model parameters and properties of the uniform steady state.

	Locus A	Locus B
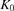	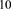	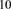
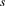	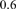	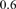
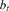	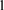	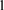
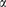	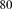	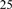
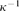	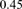	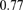
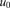 *	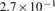	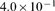
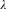	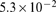	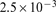
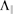 †	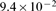	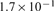
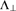	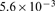	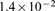

*: 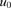 is the concentration of complexes in the unstable uniform steady state.

**†:** 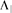 and 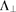 are defined in the supporting analysis.

The noise term in Eq. (4) is Gaussian with covariance
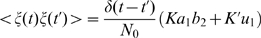
(8)To derive this relation, recall that all concentrations were rescaled so that 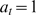. The total concentration 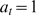 of 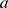 proteins corresponds to having 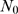 molecules per interface, by definition of 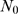. Hence the number of 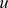 complexes on the interface is given by 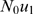 (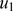 is thus the fraction of 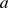 proteins that participate in a complex). Assuming Poisson statistics, the variance in the number of reactions per unit time is given by 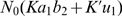 which, after division by 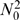 yields Eq. (8).

#### Stochastic simulations

We used a forward explicit Euler method for simulating the stochastic equations on a lattice of cells. In each step a set of 12 linear equations are solved in each cell to obtain the field 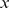 on the membrane and its average 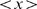 on each of the six sides. Eq. (4) is then used to update the two complex concentrations on each interface. The time step was 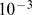. A typical simulation such as the one in Fig. 5 requires 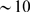 hours on an Intel Core 2 processor.

#### Global field

When analyzing the effect of graded 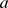 expression we use an equivalent constant field, projected onto the dipole carrying modes, as described in Text S1, part II (Eqs. S54–S55), rather than include explicitly a graded expression of 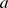, in order to avoid boundary effects. However, we also ran simulations on large lattices with direct gradients of 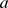 to verify the applicability of Eq. (S55). In Fig. 7 a local field was associated with each cell. The dynamic equations at each interface involved a local field taken as the average of the fields on the two cells separated by the interface.

#### Dipole moment

We define the magnitude of the PCP dipole in each cell as 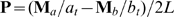 where 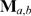 is the dipole moment of 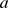/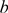 protein distribution, 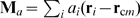 and the sum is over all sides of the cell, 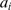 is the 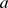 concentration on side 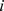, 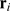 is side 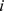
's center, and 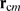 is the cell's center. A similar equation holds for 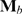. The only contribution to 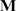 comes from the complexed proteins because the free proteins are uniformly distributed in the cell.

#### Fig 2A

For illustration purposes we set here 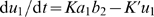 where 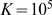, 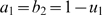, 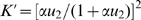 and 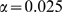.

#### Phase diagram

The phase diagram (Fig. 3) was obtained as follows. In the deterministic limit the steady states of the system are spatially uniform. Hence the problem reduces to that of finding the steady states of a six-dimensional dynamical system. The phase space was first sampled at 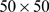 discrete loci to obtain a low-resolution representation of the phase diagram. At each point all steady states (stable and unstable) were found using a multidimensional secant root-finding algorithm as described in [38], initialized with 500 different random states. For each stable state found in this way, the stability and symmetry properties were then determined. In all cases there was a unique stable steady state up to the symmetry: Either a single stable uniform state, or six equivalent stable vertex states, or six equivalent stable side states. After obtaining the low-resolution representation of the phase diagram, we used the continuity of the phase transitions in order to obtain precise phase boundary curves, by solving numerically for loci where an eigenvalue of the Jacobian matrix vanishes.

## Supporting Information

Figure S1 Coarsening dynamics. Coarsening dynamics in a stochastic simulation without an orienting signal, at several time points: λ*t* = 2.5 (A) - before amplitude saturation, λ*t* = 10 (B), 50 (C), and 100 (D). Parameters are the same as in Fig. ∼5.
(8.34 MB TIF)

Figure S2 Effect of delayed application of the orienting field. Dashed lines show the average polarization in the distal direction when a bulk orienting signal is applied only from λ*t* = 5, compared to the dynamics when the field is applied from the simulation onset (full lines). Black, red, and gray traces correspond to three different magnitudes of the applied field. These correspond, respectively, to gradients in a concentration that amount to an increase of 0.5%, 0.2%, and 0.1% from each cell to its proximal neighbor.
(0.32 MB EPS)

Figure S3 Notation used in the dynamic equation for *u*_1_. Note that *u*_1,0_ = *u*′_2,0_≡*u*_1_ and *u*_2,0_ = *u*′_1,0_≡*u*_2_.
(0.44 MB EPS)

Text S1 Supporting text.
(0.25 MB PDF)

## References

[1] Adler PN (2002). Planar signaling and morphogenesis in drosophila.. Dev Cell.

[2] Mlodzik M (2002). Planar cell polarization: do the same mechanisms regulate drosophila tissue polarity and vertebrate gastrulation?. Trends Genet.

[3] Tree DRP, Ma D, Axelrod JD (2002). A three-tiered mechanism for regulation of planar cell polarity.. Semin Cell Dev Biol.

[4] Eaton S (2003). Cell biology of planar polarity transmission in the drosophila wing.. Mech Dev.

[5] Jones C, Chen P (2007). Planar cell polarity signaling in vertebrates.. BioEssays.

[6] Wong LL, Adler PN (1993). Tissue polarity genes of drosophila regulate the subcellular location for prehair initiation in pupal wing cells.. J Cell Biol.

[7] Strutt DI (2002). Asymmetric localization of frizzled and the establishment of cell polarity in the drosophila wing.. Mol Cell.

[8] Axelrod JD (2001). Unipolar membrane association of dishevelled mediates frizzled planar cell polarity signaling.. Gene Dev.

[9] Feiguin F, Hannus M, Moldzik M, Eaton S (2001). The ankyrin repeat protein diego mediates frizzled-dependent planar polarization.. Dev Cell.

[10] Tree DR, Shulman JM, Rousset R, Scott MP, Gubb D (2002). Prickle mediates feedback amplification to generate asymmetric planar cell polarity signaling.. Cell.

[11] Shimada Y, Usui T, Yanagawa S, Takeichi M, Uemura T (2001). Asymmetric colocalization of flamingo, a seven-pass transmembrane cadherin, and dishevelled in planar cell polarization.. Curr Biol.

[12] Bastock R, Strutt H, Strutt D (2003). Strabismus is asymmetrically localised and binds to prickle and dishevelled during drosophila planar polarity patterning.. Development.

[13] Vinson CR, Adler PN (1987). Directional non-cell autonomy and the transmission of polarity information by the frizzled gene of drosophila.. Nature.

[14] Ma D, Yang CH, McNeill H, Simon MA, Axelrod JD (2003). Fidelity in planar cell polarity signaling.. Nature.

[15] Ma D, Amonlirdviman K, Raffard RL, Abate A, Tomlin CJ (2008). Cell packing influences planar cell polarity signaling.. Proc Natl Acad Sci USA.

[16] Affolter M, Basler K (2007). The decapentaplegic morphogen gradient: from pattern formation to growth regulation.. Nat Rev Genet.

[17] Saburi S, McNeill H (2005). Organising cells into tissues: new roles for cell adhesion molecules in planar cell polarity.. Curr Opin Cell Biol.

[18] Lawrence PA, Struhl G, Casal J (2008). Do the protocadherins fat and dachsous link up to determine both planar cell polarity and the dimensions of organs?. Nat Cell Biol.

[19] Irvine K (2008). Private communication..

[20] Pathria RK (1996). Statistical Mechanics.

[21] Amonlirdviman K, Khare NA, Tree DRP, Chen WS, Axelrod JD (2005). Mathematical modeling of planar cell polarity to understand domineering nonautonomy.. Science.

[22] Swain PS, Elowitz MB, Siggia ED (2003). Intrinsic and extrinsic contributions to stochasticity in gene expression.. Proc Natl Acad Sci USA.

[23] Park WJ, Liu J, Adler PN (1994). Frizzled gene expression and development of tissue polarity in the drosophila wing.. Dev Gen.

[24] Elitzur S, Pearson RB, Shigemitsu J (1979). Phase structure of discrete abelian spin and gauge systems.. Phys Rev D.

[25] Cardy J (1980). General discrete planar models in two dimensions: Duality properties and phase diagrams.. J Phys A.

[26] Einhorn MB, Savit R, Rabinovici E (1980). A physical picture for the phase transitions in *z_n_* symmetric models.. Nucl Phys B.

[27] Kaski K, Gunton JD (1983). Universal dynamical scaling in the clock model.. Phys Rev B.

[28] Chaikin PM, Lubensky TC (1995). Principles of condensed matter physics.

[29] Hannus M, Feiguin F, Heisenberg CP, Eaton S (2002). Planar cell polarization requires widerborst, a b' regulatory subunit of protein phosphate 2a.. Development.

[30] Shimada Y, Yonemura S, Ohkura H, Strutt D, Uemura T (2006). Polarized transport of frizzled along the planar microtubule arrays in drosophila wing epithelium.. Dev Cell.

[31] Axelrod J (2008). Private communication..

[32] Classen AK, Anderson KI, Marois E, Eaton S (2005). Hexagonal packing of drosophila wing epithelial cells by the planar cell polarity pathway.. Dev Cell.

[33] Adler PN, Krasnow RE, Liu J (1997). Tissue polarity points from cells that have higher frizzled levels towards cells that have lower frizzled levels.. Curr Biol.

[34] Simizu-Sato S, Huq E, Tepperman JM, Quail PH (2002). A light-switchable promoter system.. Nature Biotech.

[35] Park WJ, Liu J, Adler PN (1994). The frizzled gene of drosophila encodes a membrane protein with an odd number of transmembrane domains.. Mech Dev.

[36] Krasnow RE, Adler PN (1994). A single frizzled protein has a dual function in tissue polarity.. Development.

[37] Tomlinson A, Ready DF (1987). Neuronal differentiation in drosophila ommatidium.. Dev Biol.

[38] Press WH, Teukolsky SA, Vetterling WT, Flannery BP (1992). Numerical Recipes in C.

[39] Usui T, Shima Y, Shimada Y, Hirano S, Burgess RW (1999). Flamingo, a seven-pass transmembrane cadherin, regulates planar cell polarity under the control of frizzled.. Cell.

[40] Adler PN, Taylor J, Charlton J (2000). The domineering non-autonomy of frizzled and van gogh clones in the drosophila wing is a consequence of a disruption in local signaling.. Mech Dev.

