# Supplementary figures and images for: Order and Stochastic Dynamics in Drosophila Planar Cell Polarity
Source: PLoS Comput Biol. 2009 Dec 24;5(12):e1000628. doi: 10.1371/journal.pcbi.1000628 (PMC2791803; doi:10.1371/journal.pcbi.1000628)

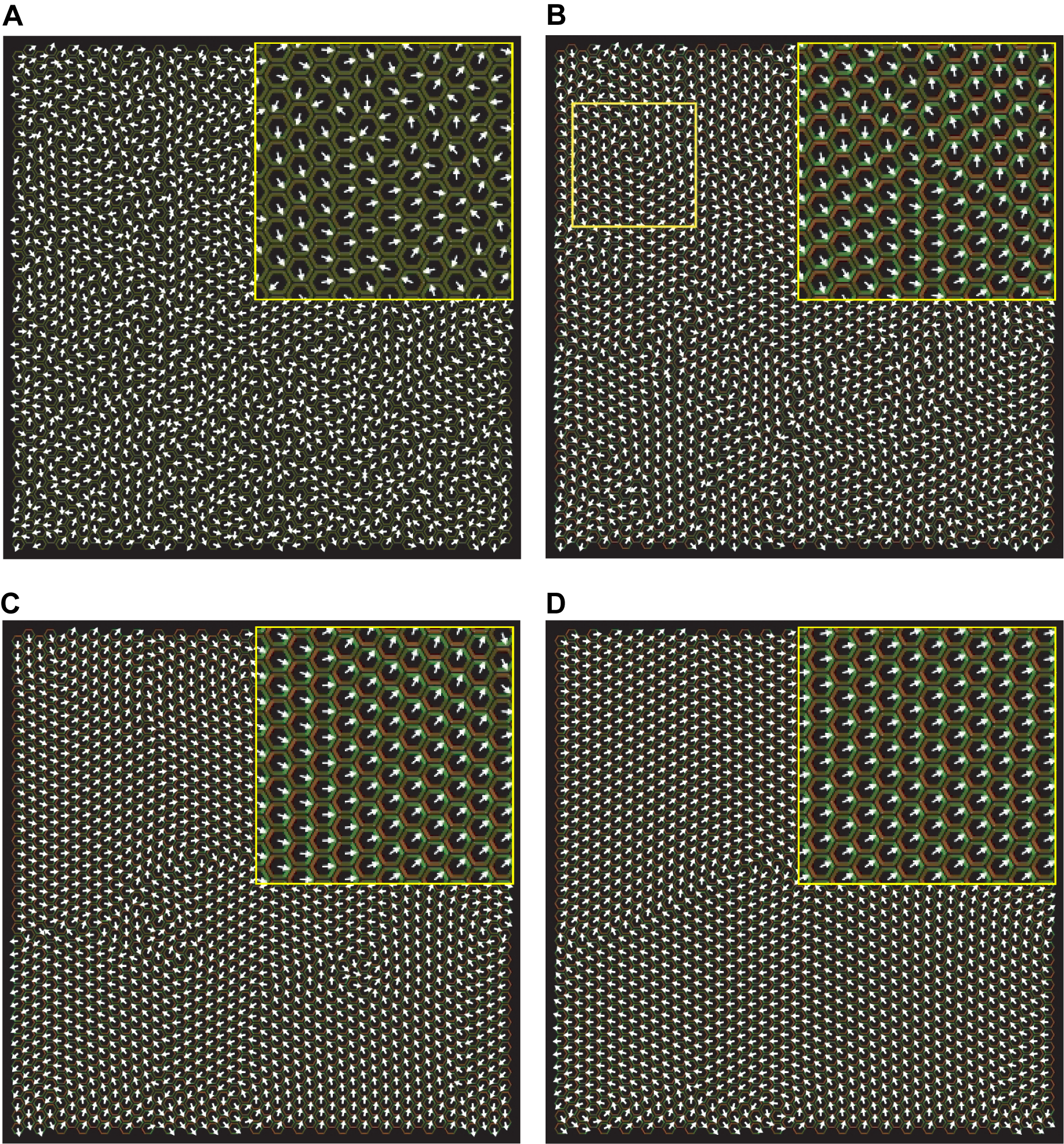

Supplement: Figure S1 — Coarsening dynamics. Coarsening dynamics in a stochastic simulation without an orienting signal, at several time points: λt = 2.5 (A) - before amplitude saturation, λt = 10 (B), 50 (C), and 100 (D). Parameters are the same as in Fig. ∼5. (8.34 MB TIF) [file pcbi.1000628.s001.tif]
