# Supplementary material for: Order and Stochastic Dynamics in Drosophila Planar Cell Polarity
Source: PLoS Comput Biol. 2009 Dec 24;5(12):e1000628. doi: 10.1371/journal.pcbi.1000628 (PMC2791803; doi:10.1371/journal.pcbi.1000628)
Supplement: Text S1 — Supporting text. (0.25 MB PDF) [file pcbi.1000628.s004.pdf]

Order and stochastic dynamics of planar cell polarity in *Drosophila*
PLoS Computational Biology: Supporting information (Text S1)

Yoram Burak

Center for Brain Science, Harvard University, Cambridge, MA 02138 and

Kavli Institute for Theoretical Physics,

University of California, Santa Barbara, CA 93106, USA

Boris I. Shraiman

Kavli Institute for Theoretical Physics and Department of Physics,

University of California, Santa Barbara, CA 93106, USA

I. MODIFICATION OF A DIFFUSIBLE PROTEIN

We discuss how the modification of a diffusible protein can generate a non-local field responding to the distribution of complexes on the membrane. We present two different scenarios, to demonstrate that many variations on this theme are possible. The second scenario, which is used in the main manuscript, is deliberately chosen to be similar to the mechanism assumed in Ref. 21. We generated phase diagrams and ran stochastic simulations in both of the scenarios. The qualitative properties discussed in the main part of the manuscript apply to both of the mechanisms.

A. Scenario 1 - Cytosolic Messenger Protein

This mechanism utilizes a messenger protein and is described schematically in Fig. 2D of the main manuscript. The messenger protein is taken to be cytosolic (a membrane-bound messenger will yield similar results.) The messenger protein binds to the b side of a-b complexes, where it is chemically modified, and is released back to the cytoplasm. For concreteness we assume that the modification is a phosphorylation. In its phosphorylated state, a messenger hitting the a side of a b-a complex promotes the unbinding of this complex. In this mechanism K' in Eq. (1) of the main manuscript increases with d, whereas K is taken as a constant.

1. *Non-locality of inhibition*

To see why inhibition is non-local we consider a quenched distribution of a–b complexes on the membrane and evaluate the position-dependent phosphorylation of the messenger protein in the cytoplasm. Concentrating on the lower side of the interface shown in Fig. 2D of the main manuscript, we denote the concentration of cytosolic proteins by $c(\mathbf{r})$, and the fraction of these proteins that are phosphorylated by $x(\mathbf{r})$. These fields are assumed to equilibrate rapidly compared to the rate at which the concentration of a–b complexes varies on the membrane. At steady state $c(\mathbf{r})$ is then uniform in the cytoplasm, and thus cannot convey any information about the complex distribution. On the other hand the value of $x(\mathbf{r})$ at contact with the membrane is obtained by solving an equation in the cytoplasm,

$$-\left(\nabla^2 + \kappa^2\right) x = 0. \tag{S1}$$

The length scale κ^{-1} sets the characteristic range of the non-local inhibition. It is equal to the typical distance that a phosphorylated messenger protein diffuses in the cytoplasm before being

de-phosphorylated: $\kappa^2 = \gamma'/D_m$, where γ' is the de-phosphorylation rate and D_m is the messenger diffusion constant.

The source for equation (S1) is determined from the distribution of complexes on the membrane:

$$-\hat{n} \cdot \nabla x = \kappa\alpha\,(1-x)\,u_2, \tag{S2}$$

where u_2 is the concentration of complexes containing a b in the lower side of the interface, and α has units of inverse concentration. Here we assume that the process is limited by the binding of cytosolic protein to a–b complexes, *i.e.*, phosphorylation and release of the phosphorylated protein to the cytoplasm are fast compared to binding (this assumption is relaxed in Sec. I C.) The coefficient α is then determined by $\kappa\alpha = k/D_m$, where k is the rate constant for binding of messenger proteins to membrane complexes. If, furthermore, binding is diffusion limited then $k \simeq l D_m$, where l is the molecular size of the messenger-complex binding site, and therefore $\alpha \simeq l/\kappa$.

If the distribution of u_2 is uniform on a planar interface, we find from Eqs. (S1) and (S2),

$$x = \frac{\alpha u_2}{1+\alpha u_2} \tag{S3}$$

Hence α sets the characteristic concentration where $x(u_2)$ saturates at unity. If u_2 complexes are not uniformly distribution on the membrane, x responds in a non-local fashion, characterized by a length scale equal (for small d) to κ^{-1}.

2. Necessity for non-equilibrium thermodynamics

The mechanism outlined above involves completely irreversible reactions, which can be relaxed by assigning backward rate constants to all the intermediate steps: (*i*) binding of messenger to complex, (*ii*) phosphorylation of the messenger, (*iii*) release of the phosphorylated messenger to the cytoplasm, and (*iv*) messenger de-phosphorylation in the cytoplasm (Fig. S4). If all processes are in thermal equilibrium a constraint is imposed on the rate constants,

$$\Gamma\gamma' k k_p' = \Gamma'\gamma k_p k' \tag{S4}$$

When this constraint is obeyed, the field $x(\mathbf{r})$ becomes uniform throughout the cytoplasm at steady state, and cannot convey any information about the non-uniform distribution of complexes on the membrane. This is shown in detail in Sec. I C.

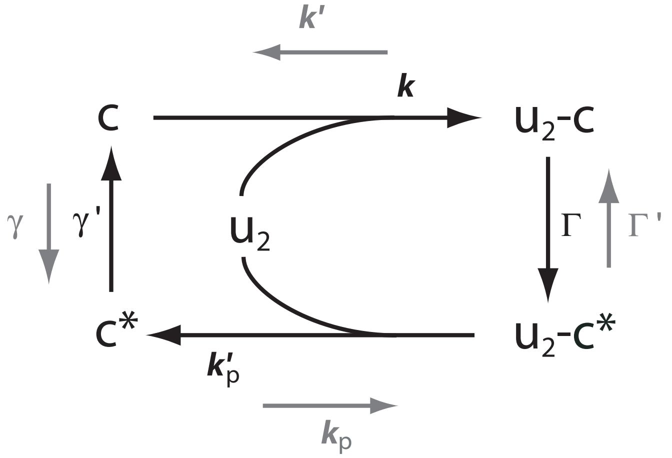

FIG. S4: **Detailed reactions in scenario 1**. The messenger c binds the b side of an a-b complex (u_2). It is then phosphorylated at a rate Γ and is released to the cytoplasm. The modified messenger diffuses in the cytoplasm, where it is spontaneously de-phosphorylated at a rate γ'. The back-reactions are considered in Sec. I C.

3. Summary of the dynamic equations in scenario 1

Because modified messengers promote the degradation of complexes, the dynamic equation, Eq. (1) in the main manuscript, takes the form

$$\begin{aligned} \frac{\mathrm{d}u_1}{\mathrm{d}t} &= K_0 \left(a_t - \sum_{i \in I} u_{1,i}/6 \right) \left(b_t - \sum_{i \in II} u_{2,i}/6 \right) \\ &- \langle x^\nu \rangle \, u_1 \end{aligned} \tag{S5}$$

where degradation is described by the second term which depends on the phosphorylation fraction x. The exponent ν represents cooperativity: an exponent $\nu > 1$ is required to obtain instability, and in practice we take $\nu = 2$ (there are other possible ways to obtain the required non-linearity: see, for example, the non-linearity in scenario 2;) $\langle x^\nu \rangle_i$ is the average of x^ν on the upper side of the interface. The field x is determined from a solution in the cytoplasm of Eqs. (S1) and (S2) where, to simplify the solution, we treat the cell as a circular object of radius $S = L/\sqrt{3}$ where S is the length of a side.

In Eq. (S5) time has been rescaled by the rate at which messenger proteins hit a b molecule, and therefore the coefficient of the second term on the right-hand side is unity. In addition we work with dimensionless concentrations, scaled by the total density of a proteins, so that $a_t = 1$. The independent parameters in the model are then K_0, b_t, ν, α, and κ.

B. Scenario 2 - Direct modification of a or b

In this scenario there is no separate messenger protein. Instead, one of the two proteins comprising the a-b complexes is modified directly (we take this to be a.) For concreteness, we assume that the nature of the modification is the binding to a of a cytosolic protein.

The mechanism is described in Fig. 2E of the main manuscript. Complexes containing b in the lower cell inhibit modification of a. This inhibition acts locally but its effect is non-local due to the diffusion of the a proteins within the membrane. Non-locality can extend from one interface to another within the same cell because the unbound a proteins are free to diffuse from one interface to another.

In this mechanism it is easy to couple either K or K' to the non-local field, as this field represents a direct modification of the complex-forming proteins. The coupling that we assume is that only modified a proteins can form a complex with a b in the neighboring cell. Therefore u_2 complexes inhibit formation of u_1 complexes, and K in Eq. (1) of the main manuscript decreases with u_2. This mechanism is similar to the one used in Ref. 21, where modification of fz (a) by binding of the cytosolic protein dsh is inhibited by vang (b).

1. *The non-local field*

We denote the rate at which a proteins are modified by k and the rate at which they are unmodified by k'. To simplify the comparison with the previous scenario, we define the local field x as the fraction of *unmodified* a proteins. With this definition a larger value of x implies a larger inhibition of complex formation, as in scenario 1. The non-local field obeys the equation

$$\left[-D\nabla_s^2 + (k + k')\right] x(\mathbf{r}_s) = k'(\mathbf{r}_s) \tag{S6}$$

where D is the diffusion constant of the membrane protein. We use the subscript 's' in ∇_s and $\mathbf{r}_s$ to denote locations confined to the cell membrane (this notation is omitted in the main text).

For simplicity we take k to be a constant. If cytosolic proteins are available at high excess and their binding to membrane complexes is diffusion limited, then $k \simeq lD_c c$, where c is the concentration of cytosolic proteins, l is the molecular size of the binding site, and D_c is the diffusion constant of cytosolic protein. We assume that unbinding of the cytosolic protein is promoted by encountering a d complex. For simplicity we assume that there is no additional, spontaneous, unbinding and therefore we take k' to be proportional to u_2. If each encounter of a modified a with

a u_2 complex results in unbinding of the cytosolic modifier, and if these encounters are diffusion limited, then $k' \sim Dd$.

It is convenient to parameterize k and k' as follows,

$$\begin{aligned} k &\equiv D\kappa^2 \\ k' &\equiv D\kappa^2 \cdot \alpha u_2 \end{aligned} \tag{S7}$$

Equation (S6) then takes the form

$$\left(-\nabla_s^2 + \kappa^2\right) x = \kappa^2 \alpha u_2 (1 - x) \tag{S8}$$

which is very similar to Eqs. (S1-S2) for x in scenario 1: If u_2 complexes are uniformly distributed on the membrane then x is given by

$$x = \frac{\alpha u_2}{1 + \alpha u_2},$$

[compare with Eq. (S3)]. If u_2 complexes are not uniformly distribution on the membrane, x responds in a non-local fashion, characterized by a length scale equal (for small u_2) to κ^{-1}.

2. *Summary of the dynamic equations in scenario 2*

As discussed earlier, we assume that modification of a promotes complex formation. We assume that only modified a-s can bind b, whereas unbinding does not depend on the modification. The dynamic equation has the following form,

$$\begin{aligned} \frac{\mathrm{d}u_1}{\mathrm{d}t} &= K_0(1 - \langle x \rangle)(1 + s u_1) \left(a_t - \sum_{i \in I} u_{1,i}/6\right) \left(b_t - \sum_{i \in II} u_{2,i}/6\right) \\ &- u_1 \end{aligned} \tag{S9}$$

where $\langle x \rangle$ is the average of x on the interface and x is determined from Eq. (S8), on the interfacial membrane of the top cell. We work with dimensionless concentrations such that $a_t = 1$, *i.e.*, the unit of concentration is the total density of a proteins. A rescaling of time is implied in Eq. (S9) since the rate constant for unbinding $K' = 1$, *i.e.*, the unit of time is the inverse rate for a-b unbinding. The parameters in the model are thus K_0, b_t, s, α, and κ.

There are alternative ways to introduce the non-linearity necessary to obtain an instability, instead of including the self-excitation term. For example, non-linearity can be achieved also if both the a and b proteins are modified. This possibility is interesting in light of the known molecular players in PCP within the wing, as both fz and vang appear to bind a cytosolic protein (Disheveled and Prickled, respectively).

C. Detailed analysis of the non-local field in Scenario 1

Here we relax several of the assumptions made in Sec. I A and analyze the non-local field in this more general setup. An important aspect of the generalization is that all the reactions can be reversible. The notation is shown Fig. S4.

We focus on the way that the fraction of modified messenger proteins, $x(\mathbf{r})$ responds at steady state to a quenched distribution of u_2 complexes, denoted $u_2(\mathbf{r}_s)$. These can exist in three states whose concentrations are denoted $u_{2,f}(\mathbf{r}_s)$ for free complexes without a bound messenger, $\hat{u}_2(\mathbf{r}_s)$ for complexes with a bound messenger, and $\hat{u}_{2,p}(\mathbf{r}_s)$ for complexes with a bound modified (phosphorylated) messenger. At every point

$$u_2(\mathbf{r}_s) = u_{2,f}(\mathbf{r}_s) + \hat{u}_2(\mathbf{r}_s) + \hat{2}_{2,p}(\mathbf{r}_s). \tag{S10}$$

Taking into account all the possible reactions in Fig. S4 we have, on the membrane,

$$\begin{aligned}
\dot{u}_{2,f} &= -kd_f c_n + k'\hat{u}_2 - k_p d_f c_p + k'_p \hat{u}_{2,p} \\
\dot{\hat{u}}_2 &= k u_{2,f} c_n - k'\hat{u}_2 - \Gamma\hat{u}_2 + \Gamma'\hat{u}_{2,p} \\
\dot{\hat{u}}_{2,p} &= k_p u_{2,f} c_p - k'_p \hat{u}_{2,p} + \Gamma\hat{u}_2 - \Gamma'\hat{u}_{2,p}
\end{aligned} \tag{S11}$$

where c_n is the concentration of unmodified messengers and c_p is the concentration of modified (phosphorylated) ones – these are three dimensional concentrations defined in the bulk, but since Eq. (S11) is defined on the membrane their value at contact with the membrane is implied. The sum $\dot{u}_{2,f} + \dot{\hat{u}}_2 + \dot{\hat{u}}_{2,p} = 0$ as it should be since we consider a quenched distribution of u_2. The partitioning of u_2 into the three possible states can be expressed in terms of the contact values of c_n and c_p,

$$\begin{pmatrix} u_{2,f} \\ \hat{u}_2 \\ \hat{u}_{2,p} \end{pmatrix} = \frac{u_2}{\alpha_1 + \alpha_2 + \alpha_3} \begin{pmatrix} \alpha_1 \\ \alpha_2 \\ \alpha_3 \end{pmatrix} \tag{S12}$$

where, using Eq. (S11),

$$\begin{aligned}
\alpha_1 &= \Gamma k'_p + (\Gamma' + k'_p)k' \\
\alpha_2 &= (\Gamma + k'_p)kc_n + \Gamma' k_p c_p \\
\alpha_3 &= \Gamma k c_n + (\Gamma + k')k_p c_p
\end{aligned} \tag{S13}$$

For the messenger proteins we have, in the cytoplasm,

$$\begin{aligned}\dot{c}_n &= D_m\nabla^2 c_n + \gamma' c_p - \gamma c_n \\ \dot{c}_p &= D_m\nabla^2 c_p + \gamma c_n - \gamma' c_p \end{aligned} \tag{S14}$$

and the boundary condition for these equations, on the membrane, is found from the reactions with the u_2 complexes,

$$\begin{aligned}-D_m\hat{\mathbf{n}}\cdot\nabla c_n &= k u_{2,f} c_n - k'\hat{u}_2 \\ -D_m\hat{\mathbf{n}}\cdot\nabla c_p &= k_p u_{2,f} c_p - k'\hat{2}_{2,p}. \end{aligned} \tag{S15}$$

Equations (S14) are diagonalized by defining

$$\begin{aligned}c_t(\mathbf{r}) &= c_n(\mathbf{r}) + c_p(\mathbf{r}) \\ \Delta(\mathbf{r}) &= \frac{\gamma' c_p - \gamma c_n}{\gamma + \gamma'} \end{aligned} \tag{S16}$$

We then have $\dot{c}_t = D_m\nabla^2 c_t$ in the cytoplasm and, using Eqs. (S16) and (S13), $-D_m\hat{\mathbf{n}}\cdot\nabla c_t = 0$ on the membrane. Hence at steady state c_t is a constant equal to c, the bulk concentration of messenger proteins. For Δ we have

$$\dot{\Delta} = -D_m\left(-\nabla^2 + \kappa^2\right)\Delta$$

in the cytoplasm where

$$\kappa^2 = \frac{\gamma+\gamma'}{D_m} \tag{S17}$$

and

$$\begin{aligned}-D_m\hat{\mathbf{n}}\cdot\nabla\Delta &= \frac{u_2}{\alpha_1+\alpha_2+\alpha_3} \times \left[\left(\Gamma k k_p' + \Gamma' k_p k'\right)\Delta\right. \\ &+ \left.\left(\frac{\Gamma'\gamma k_p k' - \Gamma\gamma' k k_p'}{\gamma+\gamma'}\right) c_t\right] \end{aligned} \tag{S18}$$

at contact with the membrane.

If all reactions obey detailed balance, Eq. (S4) holds and therefore the coefficient of c_t on the right hand side of Eq. (S18) vanishes. As there is no inhomogeneous term in this equation, the solution at steady state is then simply $\Delta = 0$ and a non-local field is not generated. We thus see that to establish a non-local field the reaction rates must deviate from detailed balance and therefore the process requires an energy flux. Although this was illustrated here for scenario A, a similar conclusion can be reached for scenario B.

In the case that $\gamma = 0$ we have

$$\Delta(\mathbf{r}) = c_p(\mathbf{r}) \equiv cx(\mathbf{r}) \tag{S19}$$

or, in other words, the non-local field Δ is the concentration of modified messengers. If, furthermore, k', Γ', and k_p also vanish, so that all reactions can occur only in one direction, Eq.(S18) can be re-written as follows,

$$-D_m \hat{\mathbf{n}} \cdot \nabla x = \frac{u_2}{\alpha_1 + \alpha_2 + \alpha_3} \Gamma k k'_p (1 - x) \tag{S20}$$

and

$$\begin{aligned} \alpha_1 &= \Gamma k'_p \\ \alpha_2 &= k k'_p c(1 - x) \\ \alpha_3 &= \Gamma k c(1 - x). \end{aligned} \tag{S21}$$

The more simple Eq. (S2) is obtained in the limit where both $k'_p \gg kc$ and $\Gamma \gg kc$, *i.e.*, phosphorylation and release to the cytoplasm are instantaneous in comparison with messenger binding to the u_2 complexes, which is then the limiting step in the process.

II. LINEAR INSTABILITY

The initial dynamics of protein localization, starting from a state were proteins are uniformly distributed on the cell membrane, can be described using a linear expansion around the unstable uniform stead state.

To derive this expansion it is convenient to center the basic unit cell of the lattice at a vertex of the honeycomb lattice, as shown in Fig. S5 (note that only half the vertices participate). Each unit cell has six degrees of freedom associated with it, namely, complex concentrations (of both polarizations) on the three interfaces joined at the vertex. We denote these as $u_{\pm 1} \dots u_{\pm 3}$ as shown in the Figure.

The dynamic equations for each vertex then involve the degrees of freedom associated with that vertex as well as its six nearest neighbors, as demonstrated in Fig. S5. Writing the dynamic equation for one degree of freedom, say u_1, determines the equations for the other five due to symmetry.

The uniform steady state, in which complexes occur with the same concentration u_0 on all the interfaces and in both polarities, is determined from:

$$K\{u_0\}(a_t - u_0)(b_t - u_0) - K'\{u_0\}u_0 = 0, \tag{S22}$$

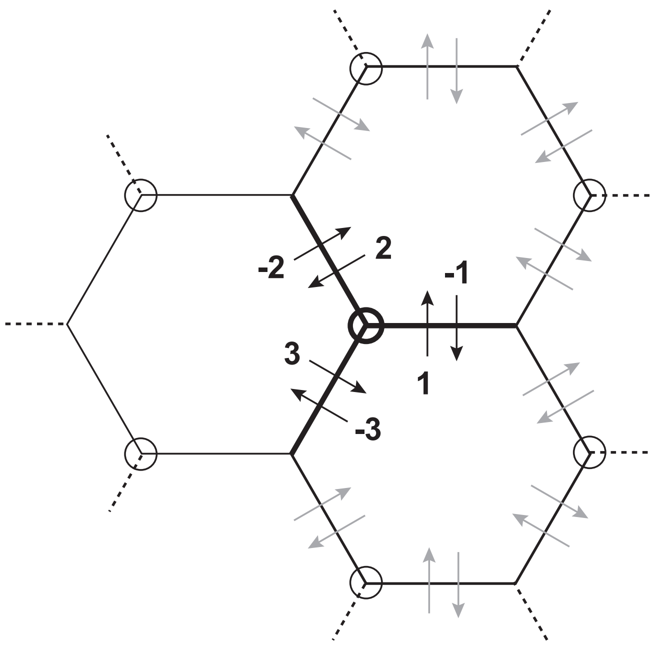

FIG. S5: **A node of the hexagonal lattice and its six nearest-neighbors**. The six degrees of freedom associated with the central node (black circle) are designated by black arrows, and the surrounding nodes are designated by thin circles. The dynamic equations for the degrees of freedom ± 1 involve the degrees of freedom corresponding to the black and gray arrows.

where $K\{u_0\}$, $K_0'\{u_0\}$ are the rate constants evaluated in the case that all the contributing complex concentrations are equal to u_0. We consider small perturbations around this steady state,

$$u_i(\mathbf{r}_\alpha) = u_0 + v_i(\mathbf{r}_\alpha) \tag{S23}$$

and expand the dynamic equations to first order. The expansion involves, in the most general situation allowed by the symmetries, 14 parameters:

$$\begin{aligned}\frac{\mathrm{d}v_0}{\mathrm{d}t} &= \alpha_0 v_0 + \alpha_1(v_1 + v_5) + \alpha_2(v_2 + v_4) + \alpha_3 v_3 \\ &+ \bar{\alpha_1}(\bar{v}_1 + \bar{v}_5) + \bar{\alpha}_2(\bar{v}_2 + \bar{v}_4) + \bar{\alpha}_3 \bar{v}_3 \\ &+ \beta_0 v_0' + \beta_1(v_1' + v_5') + \beta_2(v_2' + v_4') + \beta_3 v_3' \\ &+ \bar{\beta}_1(\bar{v}_1' + \bar{v}_5') + \bar{\beta}_2(\bar{v}_2' + \bar{v}_4') + \bar{\beta}_3 \bar{v}_3'\end{aligned} \tag{S24}$$

where we use the indexing notation shown in Fig. S6: Degrees of freedom in the lower cell are represented by v_i, v_i', and degrees of freedom in the upper cell by $\bar{v}_i$, $\bar{v}_i'$. The α coefficients are associated with complexes that share a similar polarity as that of v_0, having an a in the lower cell or a b in the upper cell. The β coefficients are associated with complexes of the opposite polarity. The notation used in Eq. (S24) can be readily translated back to the lattice indexing notations used

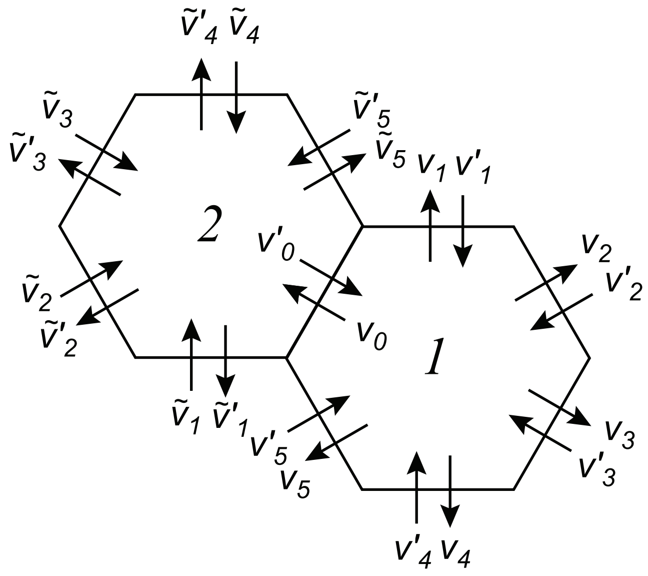

FIG. S6: **Notation used in the linear instability analysis**. Notation in Eq. (S24). The quantities v_i, v'_i (cell 1) and $\tilde{v}_i$, $\tilde{v}'_i$ (cell 2) represents deviations from the concentration in the unstable uniform steady state. Unprimed symbols are used for complexes with the same polarization as v_0, whereas primed symbols are used for opposite polarization.

in Fig. S5. For any particular realization of the model these parameters follow in a straightforward manner from the dynamic equation [*e.g.* Eq. (S9).]

A. Spatially uniform perturbation

We first consider the stability of the uniform state to perturbations that are spatially uniform, $u_i(\mathbf{r}_\alpha) = u_0 + v_i$ for all α. In these states exchanging the complex concentrations of opposite polarities on each interface is a symmetry operation. The eigenstates are thus either symmetric or antisymmetric: In the notation of Fig. S5, $v_i = v_{-i}$ or $v_i = -v_{-i}$, respectively. Since we expect the dynamics to produce bistability, the interesting situation is that in which antisymmetric modes are unstable.

The eigenstates of the linear time evolution operator are shown in Fig. S7 and follow directly from the symmetry, *i.e.*, they do not depend on the values of the coefficients α_i and β_i. There are three antisymmetric modes and three symmetric modes. For brevity we list here only the components $v_{1,2,3}$ of these states, using the notation of Fig. S5 (the other three, $v_{-1,-2,-3}$, follow

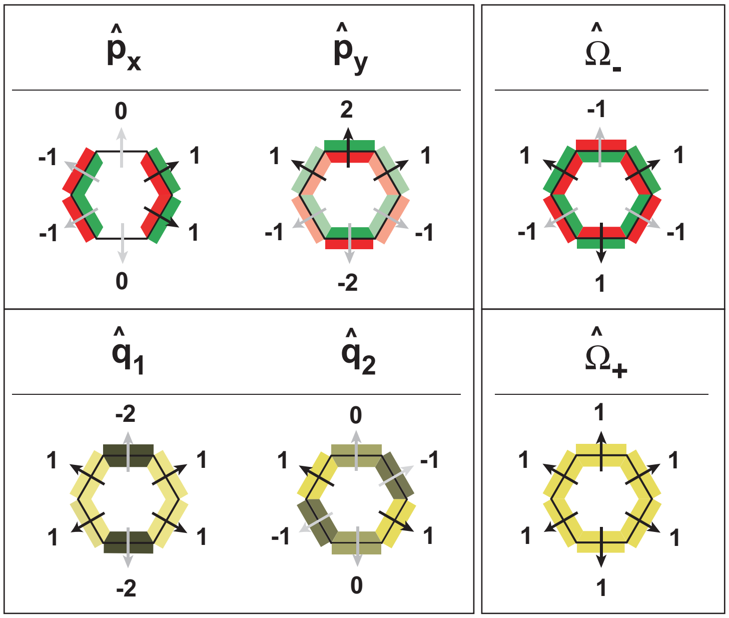

FIG. S7: **Uniform linear modes**. Structure of the spatially uniform linear modes, Eqs. (S25)–(S26).

from symmetry.) The Antisymmetric modes are:

$$\hat{p}_x : \frac{1}{2}\begin{pmatrix} 0 \\ -1 \\ 1 \end{pmatrix} \quad ; \quad \hat{p}_y : \frac{1}{2\sqrt{3}}\begin{pmatrix} 2 \\ -1 \\ -1 \end{pmatrix} \quad ; \quad \hat{\Omega}_- : \frac{1}{\sqrt{6}}\begin{pmatrix} 1 \\ 1 \\ 1 \end{pmatrix} \tag{S25}$$

The first two are degenerate (any linear combination of them is an eigenstate with the same eigenvalue). The symmetric modes are:

$$\hat{q}_1 : \frac{1}{2\sqrt{3}}\begin{pmatrix} -2 \\ 1 \\ 1 \end{pmatrix} \quad ; \quad \hat{q}_2 : \frac{1}{2}\begin{pmatrix} 0 \\ -1 \\ 1 \end{pmatrix} \quad ; \quad \hat{\Omega}_+ : \frac{1}{\sqrt{6}}\begin{pmatrix} 1 \\ 1 \\ 1 \end{pmatrix} \tag{S26}$$

The first two of these are degenerate.

Only the antisymmetric p modes carry polarization. This can be seen by expressing the dipole moment of protein distribution, on the cell membrane, in terms of the polarization vector $\hat{v}$,

$$\mathbf{M}_a = -\mathbf{M}_b = \frac{L\sqrt{3}}{2}(p_x\hat{\mathbf{x}} + p_y\hat{\mathbf{y}}) \tag{S27}$$

where $\mathbf{M}_{a,b}$ are the dipole moments of a and b proteins (as defined in *Methods*), and p_x,p_y are the $\hat{p}_x$, $\hat{p}_y$ components of $\hat{v}$, expanded in the eigenstate basis.

The polarity mode $\hat{p}_x$ points distally and has a similar structure as that of the steady-state zig-zag pattern. However at the level of the linear instability there is nothing special in a vertex

direction: all linear combinations of $\hat{p}_x$ and $\hat{p}_y$ are equivalent, and can point in any possible direction in the plane.

The eigenvalues corresponding to the p, q, and $\Omega_\pm$ modes are:

$$\begin{aligned}
\lambda_p &= \alpha_0 - \beta_0 \\
&+ \alpha_1 - \beta_1 - (\alpha_2 - \beta_2) - (\alpha_3 - \beta_3) + TC \\
\lambda_{\Omega_-} &= \alpha_0 - \beta_0 \\
&- 2(\alpha_1 - \beta_1) + 2(\alpha_2 - \beta_2) - (\alpha_3 - \beta_3) + TC \\
\lambda_q &= \alpha_0 + \beta_0 \\
&- (\alpha_1 + \beta_1) - (\alpha_2 + \beta_2) + \alpha_3 + \beta_3 + TC \\
\lambda_{\Omega_+} &= \alpha_0 + \beta_0 \\
&+ 2(\alpha_1 + \beta_1) + 2(\alpha_2 + \beta_2) + \alpha_3 + TC
\end{aligned} \tag{S28}$$

where TC represents the same set of terms, coming from complexes in the top cell instead of the bottom cell, $\alpha_i \to \bar{\alpha}_i$ and $\beta_i \to \bar{\beta}_i$ for $i = 1,2,3$. In the main manuscript we refer to λ_p simply as λ.

We make two short comments based on Eqs. (S28). First, these equations demonstrate the importance of a non-local interaction between each interface and its neighbors. If there is no such interaction, $\lambda_p = \lambda_{\Omega_-}$ and therefore the Ω_- mode grows in the same rate as the polarity carrying modes.

Second, these equations show that a mechanism based on interactions between same-polarity complexes, instead of opposite-polarity complexes ($\beta_i = \bar{\beta}_i = 0$) can, in principle, yield the desired type of instability – in contrast to the case of a single planar interface, where opposite-polarity interaction is necessary. For example, If α_1 is positive while α_2 is negative it is possible to have $\lambda_p > 0$ and $\lambda_{\Omega_-,q,\Omega_+} < 0$. This, however, requires a relatively elaborate form of the non-local interaction, involving excitation at short-range within the cell and inhibition at longer range.

B. Non-uniform perturbations

We next consider a general spatially-dependent perturbation. Due to translational symmetry the eigenstates have the form

$$u_i(\mathbf{r}_\alpha) = u_0 + v_i e^{i\mathbf{k}\cdot\mathbf{r}_\alpha}. \tag{S29}$$

where the wave-vector k is within the first Brillouin zone of the reciprocal hexagonal lattice.

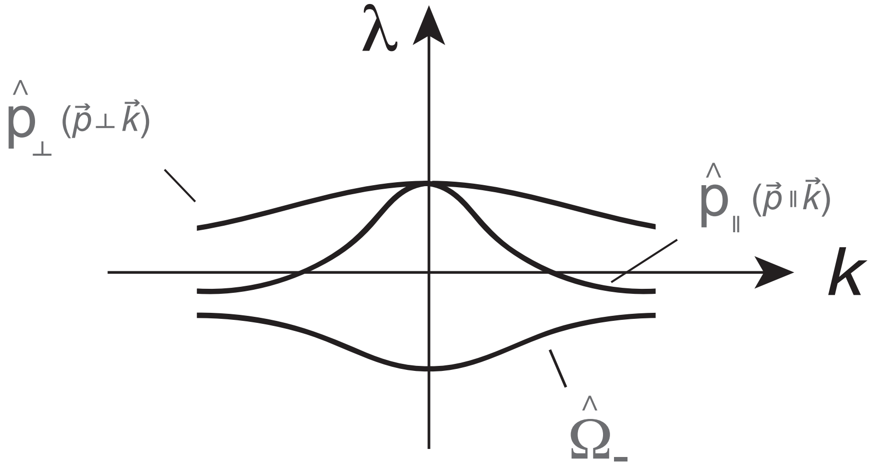

FIG. S8: **Schematic dependence of the growth rate λ on the wave-vector** k. The dependence is shown in the three antisymmetric bands $p_\perp$, $p_\parallel$, and Ω_-, in a state where planar cell polarity develops.

Since there are six degrees of freedom associated with each node, there are six bands in the $\mathbf{k}$ dependence. The two p modes, which are degenerate when $\mathbf{k} = 0$, split into two distinct bands. This is shown schematically in Fig. S8.

As we are particularly interested in the situation where only small k (large wavelength) perturbations are unstable, we look at the k dependence of the eigenvalues close to $k = 0$, which can be evaluated analytically. The most important feature of this dependence is that the eigenvalues depend only on $k_x^2 + k_y^2$, *i.e.*, the model is completely isotropic at the level of linear instability (up to 6-th order in k). Therefore the initial dynamics of polarity growth, starting from a uniform state, has no preference for any particular direction of the dipoles. Such preference arises only later on, due to nonlinear effects, as demonstrated by the existence of either a vertex-directed or side-directed stable steady-state (*Phase diagram* in the main manuscript.)

The two p bands split according to the polarity direction, one having polarization parallel to $\mathbf{k}$,

$$\hat{p}_\parallel = \frac{k_x \hat{p}_x + k_y \hat{p}_y}{|\mathbf{k}|}, \tag{S30}$$

the other with polarization transverse to $\mathbf{k}$,

$$\hat{p}_\perp = \frac{k_y \hat{p}_x - k_x \hat{p}_y}{|\mathbf{k}|}. \tag{S31}$$

For small k

$$\begin{aligned} \lambda_\parallel &= \lambda_p - \Lambda_\parallel k^2 \\ \lambda_\perp &= \lambda_p - \Lambda_\perp k^2 \end{aligned} \tag{S32}$$

where $\Lambda_{\|}$, $\Lambda_{\perp}$ depend on the α_i, β_i:

$$
\begin{aligned}
\Lambda_{\|} &= \frac{1}{8}\left(-3\alpha_2+3\beta_2-3\alpha_3+3\beta_3+TC\right)-\frac{\left(\alpha_1-\beta_1-\alpha_3+\beta_3-TC\right)\left(\alpha_1+\beta_1-\alpha_3-\beta_3-TC\right)}{8\left(\alpha_1-\alpha_3-\beta_0+\beta_2+TC\right)} \\
&+ \frac{\left(\alpha_1-\beta_1+3\alpha_2-3\beta_2+2\alpha_3-2\beta_3-TC\right)\left(\alpha_1+\beta_1+3\alpha_2+3\beta_2+2\alpha_3+2\beta_3-TC\right)}{8\left(\alpha_1+3\alpha_2+2\alpha_3+2\beta_0+3\beta_1+\beta_2+TC\right)} \\
\Lambda_{\perp} &= \frac{1}{8}\left(\alpha_1-\beta_1-\alpha_3+\beta_3+TC\right)-\frac{\left(\alpha_1-\beta_1-\alpha_3+\beta_3-TC\right)\left(\alpha_1+\beta_1-\alpha_3-\beta_3-TC\right)}{8\left(\alpha_1-\alpha_3-\beta_0+\beta_2+TC\right)} \qquad \text{(S33)}
\end{aligned}
$$

and similar expressions can be written for the other four bands. To obtain the correct type of instability both these coefficients should be positive. This is the typical situation in the models that we consider here when the p modes are unstable. Furthermore, typically we have $|\Lambda_{\|}| \gg |\Lambda_{\perp}|$ so that parallel modes are more suppressed than the transverse ones – explaining the swirling patterns generated in the absence of an orienting field: in the extreme limit where $|\Lambda_{\|}/\Lambda_{\perp}| \to \infty$ the divergence of the orientation field must vanish.

C. Strictly local interactions

It is instructive to look at the situation where all the interactions are local. The rate constants K and K' are then local functions of the complex concentrations u and d. By comparing Eqs. (1) and (S24) it is easy to see that

$$
\begin{aligned}
\alpha_0 &= \left.\frac{\partial K}{\partial u}\right|_{u_0} a_0 b_0 - \left.\frac{\partial K'}{\partial d}\right|_{u_0} u_0 - K'_0 \frac{1}{6} K_0 (a_0+b_0) \\
\alpha_{1,2,3} &= -\frac{1}{6} K_0 b_0 \\
\bar{\alpha}_{1,2,3} &= -\frac{1}{6} K_0 a_0 \\
\beta_0 &= \left.\frac{\partial K}{\partial d}\right|_{u_0} a_0 b_0 - \left.\frac{\partial K'}{\partial d}\right|_{u_0} u_0 \\
\beta_{1,2,3} &= \bar{\beta}_{1,2,3} = 0 \qquad \text{(S34)}
\end{aligned}
$$

and from Eq. (S33),

$$
\begin{aligned}
\Lambda_{\|} &= \frac{1}{8} K_0 (a_0+b_0) \\
\Lambda_{\perp} &= 0 \qquad \text{(S35)}
\end{aligned}
$$

This result, in addition to the fact that $\lambda_p = \lambda_{\Omega_-}$, demonstrates a second advantage of having non-local interactions within the cell: In the purely local case $\Lambda_{\perp} = 0$ and there is no repression of spatially varying transverse modes.

It is also interesting to look at the particular case where K and K' have no dependence on u and d or, in other words, complex formation is governed by simple binding-unbinding dynamics with no feedback. It is easy to see from Eq. (S28) that the uniform steady state is then stable (all λ-s are negative.) An orienting boundary can still induce polarity in near-by cells, and the values of λ_p, $\Lambda_\parallel$ and $\Lambda_\perp$ determine how this induced polarity is propagated. Because $\Lambda_\perp = 0$ there is no propagation in the transverse direction, except for a possible weak propagation close to the non-linearity at the boundary. In the parallel direction, however, the perturbation is propagated with a characteristic range given by $|\Lambda_\parallel/\lambda_p|^{1/2}$ in units of the cell-cell distance. With constant K and K', $\lambda_p = -K'$ and, using Eq. (S35),

$$\left|\frac{\Lambda_\parallel}{\lambda_p}\right|^{1/2} = \left[\frac{K(a_0+b_0)}{8K'}\right]^{1/2} = \left[\frac{u_0(a_0+b_0)}{8a_0b_0}\right]^{1/2} \tag{S36}$$

In the limit of strong binding, where $K/K' \to \infty$, a_0 or b_0 become small and the typical distance for propagation becomes large. The physical reason for the parallel propagation of polarity is here due strictly to the conservation of a and b proteins in each cell: a high concentration on one side of the cell creates a low concentration on the opposite side.

The prediction that binding of two proteins can induce spatial propagation of a polarity-like signal suggests a possible evolutionary scenario for the emergence of the PCP pathway. In certain tissues it may be useful to have a finite propagation of a polarity signal from a boundary, over a finite number of cells. This can be achieved using a relatively simple mechanism, involving two proteins that form complexes across inter-cellular interfaces. A more elaborate mechanism could have arisen as a refining step, involving an internal feedback mechanism within the cells, which creates an instability that amplifies the polarity signal. Finally, the PCP pathway may have been further refined by making the feedback act non-locally within each cell.

D. Dynamics

In this section we return to the case where the polarity modes are unstable, and study the dynamics – starting at $t = 0$ from the uniform steady state where all $u_i(\mathbf{r})_\alpha = u_0$. The noise variance ξ_0^2 in this state is

$$\xi_0^2 = \frac{1}{N_0}\left(K_0a_0b_0 + K_0'u_0\right) = \frac{2K_0'u_0}{N_0} \tag{S37}$$

where $a_0 = a_t - u_0$, $b_0 = b_t - u_0$, and K_0, K_0', and u_0 are the values of K,K' and u in the uniform stead state.

Although the analysis here applies in the strict sense only to this particular initial condition, we also ran simulations starting from an initial state where there are no complexes, $u_\alpha(\mathbf{r}_i) = 0$. The conclusions on amplitude and orientation dynamics were practically the same in both cases. The reason for this is that complexes first form rapidly in a symmetric manner, bringing the system close to the symmetric unstable steady state.

1. No global signal

The dynamics of a mode with eigenvalue λ, developing in time according to

$$\frac{\mathrm{d}}{\mathrm{d}t}a = \lambda a + \xi, \tag{S38}$$

where

$$\big\langle \xi(t)\xi(t')\big\rangle = \xi_0^2\delta(t-t') \tag{S39}$$

is given by

$$\big\langle a^2(t)\big\rangle = \frac{\xi_0^2}{2\lambda}(\mathrm{e}^{2\lambda t}-1) \tag{S40}$$

From this relation it is possible to calculate the spatial correlation function of the order parameter,

$$\begin{aligned}\langle(\mathbf{P}(\mathbf{r}_1)\cdot\hat{\mathbf{u}}_1)(\mathbf{P}(\mathbf{r}_2)\cdot\hat{\mathbf{u}}_2)\rangle &= \frac{1}{|\det\hat{M}|}\frac{3}{16}\left(\frac{1}{a_t}+\frac{1}{b_t}\right)^2 \\ \times\xi_0^2\int \mathrm{d}\mathbf{k}\,\mathrm{e}^{i\mathbf{k}\cdot(\mathbf{r}_1-\mathbf{r}_2)} &\left\{\frac{\mathrm{e}^{2\lambda_\parallel(k)t}-1}{2\lambda_\parallel(k)}(\hat{\mathbf{p}}_\parallel\cdot\hat{\mathbf{u}}_1)(\hat{\mathbf{p}}_\parallel\cdot\hat{\mathbf{u}}_2)\right. \\ &\left.+\frac{\mathrm{e}^{2\lambda_\perp(k)t}-1}{2\lambda_\perp(k)}(\hat{\mathbf{p}}_\perp\cdot\hat{\mathbf{u}}_1)(\hat{\mathbf{p}}_\perp\cdot\hat{\mathbf{u}}_2)\right\}\end{aligned} \tag{S41}$$

where the integration is within the unit cell of the reciprocal lattice, whose area is $|\det\hat{M}|$,

$$\frac{1}{|\det\hat{M}|} = \frac{1}{(2\pi)^2}\frac{\sqrt{3}}{2}, \tag{S42}$$

$\mathbf{p}_\parallel$ and $\mathbf{p}_\perp$ are defined in Eqs. (S30)-(S31), and Eqs. (S27) and (14) were used to express the order parameter $\mathbf{P}$ using the amplitudes of the polarity modes.

We first evaluate explicitly $\big\langle P_x^2(\mathbf{r})\big\rangle$, which is also equal to $\big\langle P_y^2\big\rangle$ due to isotropy, and corresponds in Eq. (S41) to $\hat{\mathbf{u}}_1 = \hat{\mathbf{u}}_2 = \hat{\mathbf{x}}$ and to $\mathbf{r}_1 = \mathbf{r}_2$:

$$\begin{aligned}\big\langle P_x^2\big\rangle \;\sim\; \xi_0^2\int k\mathrm{d}k\mathrm{d}\theta &\left[\cos^2\theta\cdot\frac{\mathrm{e}^{2(\lambda_p-\Lambda_\parallel k^2)t}-1}{2(\lambda_p-\Lambda_\parallel k^2)}\right. \\ &\left.+\;\sin^2\theta\cdot\frac{\mathrm{e}^{2(\lambda_p-\Lambda_\perp k^2)t}-1}{2(\lambda_p-\Lambda_\perp k^2)}\right],\end{aligned} \tag{S43}$$

where we approximated $\lambda_{\parallel,\perp}(k)$ using Eq. (S32). The leading behavior for large t is

$$\left\langle P_x^2(t)\right\rangle \simeq \frac{3\sqrt{3}}{1024\pi}\left(\frac{1}{a_t}+\frac{1}{b_t}\right)^2 \times \frac{\xi_0^2}{\lambda_p \Lambda t}\,\mathrm{e}^{2\lambda_p t} \tag{S44}$$

where

$$\Lambda \equiv \left(\frac{1}{\Lambda_\parallel}+\frac{1}{\Lambda_\perp}\right)^{-1} \tag{S45}$$

By setting $\hat{\mathbf{u}}_1 = \hat{\mathbf{u}}_2 \equiv \hat{\mathbf{u}}$ and choosing $\mathbf{r}_2 - \mathbf{r}_1$ to be parallel or perpendicular to $\hat{\mathbf{u}}$ we can evaluate from Eq. (S41) the correlation function in the parallel or perpendicular direction to the polarity vector. For large $|\mathbf{r}_2-\mathbf{r}_1|$ the parallel correlation function decays as $\exp(-r^2/8\Lambda_\parallel t)$ and the perpendicular correlation function decays as $\exp(-r^2/8\Lambda_\perp t)$. From Eq. (S44) the dipole moment saturates when

$$t \sim \frac{1}{\lambda_p}\log\frac{C}{\xi_0^2} \tag{S46}$$

where C is approximately constant. Hence we can roughly estimate the characteristic range of correlation at amplitude saturation as

$$\left(\frac{\Lambda_\parallel}{\lambda_p}\log\frac{C}{\xi_0^2}\right)^{1/2} \quad , \quad \left(\frac{\Lambda_\perp}{\lambda_p}\log\frac{C}{\xi_0^2}\right)^{1/2} \tag{S47}$$

in the parallel and perpendicular directions, respectively.

2. *Boundary signal*

In the presence of a strong orienting boundary there is inevitably a region, close to the boundary, where the linear theory is not valid. Nevertheless we can estimate the deterministic effect of the boundary by assuming a state at $t = 0$ where

$$\mathbf{P}_x(x) = A\delta(x), \tag{S48}$$

then propagating this state in time using the bulk dynamics coming from the linear instability analysis. The parameter A depends on the non-linearities close to the surface and is taken in Fig. 6 (main manuscript) from the actual value of the order parameter in the layer of cells in direct contact with the boundary. We then have

$$\begin{aligned}\langle P_x\rangle &\simeq \frac{A}{2\pi}\int \mathrm{d}k\,\mathrm{e}^{ikx}\mathrm{e}^{(\lambda_p-\Gamma_\parallel k^2)t} \\ &= A\sqrt{\frac{1}{4\pi\Lambda_\parallel t}}\exp\left(-\frac{x^2}{4\Lambda_\parallel t}\right)\exp(\lambda_p t)\end{aligned} \tag{S49}$$

To identify the position of the front, up to which all cells are expected to be aligned in the orientation set by the boundary, we compare the deterministic value of the order parameter from Eq. (S49) with the noise-driven standard deviation in the bulk from Eq. (S44), requiring: $\langle P_x(x)\rangle^2 \gtrsim \langle P_x^2(x)\rangle$, which yields

$$x \lesssim \left[2\Lambda_{\|} t \log\left(\frac{64}{3\sqrt{3}} \frac{\lambda_p \Lambda A^2}{\Lambda_{\|}\xi_0^2}\right)\right]^{1/2} \tag{S50}$$

where we assumed, for simplicity, that $b_t = a_t = 1$.

To understand the increase in propagation close to the phase boundary, note that at the time of amplitude saturation $\lambda_p t$ is of order unity (with a logarithmic correction,) and therefore $x \sim (\Lambda_{\|}/\lambda_p)^{1/2}$ up to a logarithmic prefactor. This quantity diverges at the phase boundary because $\lambda_p \to 0$ while $\Lambda_{\|}$ remains finite.

3. Bulk signal

The general framework for analyzing a weak perturbation in the bulk is as follows. Consider a set of dynamics equations for the variables $\{u_i\}$,

$$\frac{\mathrm{d}}{\mathrm{d}t} u_i = F_i(\alpha, \{u_j\}) \tag{S51}$$

where α is a control parameter for the perturbation, such that $\alpha = 0$ in the non-perturbed case. Suppose that $u_i = u_i^0$ is the steady state of Eqs. (S51) when $\alpha = 0$. We expand $u_i = u_i^0 + v_i$ and consider the dynamic equations for v_i. To first order in α

$$\begin{aligned} \frac{\mathrm{d}}{\mathrm{d}t} v_i &= \sum_j \frac{\partial F_i(0,\{u_i,0\})}{\partial u_j} v_j \\ &+ \alpha \left\{ \frac{\partial F_i(0,\{u_i,0\})}{\partial \alpha} + \sum_j \frac{\partial^2 F_i(0,\{u_i,0\})}{\partial \alpha \partial u_j} v_j \right\} \end{aligned} \tag{S52}$$

The first term is the linearized dynamics in the non-perturbed case. In the curly brackets the first term acts as a non-homogeneous driving field, while the second term becomes important only to second order in α when starting from the state $u_i = u_i^0$.

A gradient in the expression of a contributes to the driving term through the dependence of a_t on position. We consider here the case of a uniform gradient, $a_t = a_t^0 + \alpha \nabla a_t \cdot \mathbf{r}$. In a particular node on the lattice (Fig. S5) at position $\mathbf{r}$, the driving field acting on the six degrees of freedom is

given by

$$
\begin{aligned}
\mathbf{h}(\mathbf{r}) &= K(b_t - u_0)[(\nabla a_t \cdot \mathbf{r}) \\
&+ (\nabla_x a_t)\frac{L}{2\sqrt{3}}\begin{pmatrix} 1 \\ 1 \\ -2 \\ 1 \\ -2 \\ 1 \end{pmatrix} + (\nabla_y a_t)\frac{L}{2}\begin{pmatrix} -1 \\ 1 \\ 0 \\ 1 \\ 0 \\ -1 \end{pmatrix}]
\end{aligned} \tag{S53}
$$

The first term couples only to the stable symmetric modes. The second and third terms couple only to the uniform ($k = 0$) modes. Projecting them on the unstable polarity modes, Eq. (S25), (and setting $\alpha = 1$,) yields

$$
\frac{\mathrm{d}}{\mathrm{d}t}\mathbf{p}_0 = \frac{\mathrm{d}}{\mathrm{d}t}\mathbf{p}_0\bigg|_0 + \mathbf{h} \tag{S54}
$$

where $\mathbf{p}_0$ is the magnitude of the spatially uniform ($k = 0$) polarity modes, the first term on the right hand side is the derivative of $\mathbf{p}_0$ in the absence of graded expression, and

$$
\mathbf{h} = \frac{\sqrt{3}}{2}(b_t - u_0)K(\{u_0\})L\nabla a_t \tag{S55}
$$

While the above derivation describes the deterministic component in the dynamics of p_0, all spatial-frequency components with wave-vectors $\mathbf{k}$ evolve stochastically according to the equations of Secs. B and D1. Proximity to the phase boundary assists in orienting by a bulk signal because the range of wave-vectors of unstable modes around $k = 0$ becomes small, filtering out the contribution of noise at spatial frequencies other than 0.

Phase boundaries. Proximity to the phase boundary assists in orienting by a bulk signal, as discussed in the main manuscript, because the range of wave-vectors of unstable modes around $k = 0$ becomes small, filtering out the contribution of noise at large spatial frequencies.
